# Plant Secondary Metabolite Transporters: Diversity, Functionality, and Their Modulation

**DOI:** 10.3389/fpls.2021.758202

**Published:** 2021-10-27

**Authors:** Panchsheela Nogia, Pratap Kumar Pati

**Affiliations:** Department of Biotechnology, Guru Nanak Dev University, Amritsar, India

**Keywords:** ABC, MATE, metabolite transporter, NPF, PUP, secondary metabolite, systemic metabolic engineering, transporter engineering

## Abstract

Secondary metabolites (SMs) play crucial roles in the vital functioning of plants such as growth, development, defense, and survival via their transportation and accumulation at the required site. However, unlike primary metabolites, the transport mechanisms of SMs are not yet well explored. There exists a huge gap between the abundant presence of SM transporters, their identification, and functional characterization. A better understanding of plant SM transporters will surely be a step forward to fulfill the steeply increasing demand for bioactive compounds for the formulation of herbal medicines. Thus, the engineering of transporters by modulating their expression is emerging as the most viable option to achieve the long-term goal of systemic metabolic engineering for enhanced metabolite production at minimum cost. In this review article, we are updating the understanding of recent advancements in the field of plant SM transporters, particularly those discovered in the past two decades. Herein, we provide notable insights about various types of fully or partially characterized transporters from the ABC, MATE, PUP, and NPF families including their diverse functionalities, structural information, potential approaches for their identification and characterization, several regulatory parameters, and their modulation. A novel perspective to the concept of “Transporter Engineering” has also been unveiled by highlighting its potential applications particularly in plant stress (biotic and abiotic) tolerance, SM accumulation, and removal of anti-nutritional compounds, which will be of great value for the crop improvement program. The present study creates a roadmap for easy identification and a better understanding of various transporters, which can be utilized as suitable targets for transporter engineering in future research.

## Introduction

The transport of metabolites in the plants is an indispensable phenomenon for the dynamic functioning of numerous cellular processes ranging from growth, development, survival, defense, maintaining homeostasis, and coordination in the system (Larsen et al., 2017a; Erb and Kliebenstein, 2020). Specifically, the vast number of secondary metabolites (SMs) produced by plants serve crucial purposes including their conclusively proven function as defense signaling molecules for protecting against several pathogens and additionally as bioactive compounds for medicinal usage (Isah, 2019). Until recently SM transporters are being overlooked for the improvement of various biotechnological processes such as enhanced production of bioactive compounds that would provide ample supply for herbal formulations at the industrial level. Although, a few studies (Rischer et al., 2013; Gupta et al., 2017; Rastegari et al., 2019) have attempted to examine as well as regulate the functions of several key enzymes from SM biosynthetic pathways for improving the yield of bioactive compounds, but the success rate remains limited. However, in the recent past, the engineering of SM transporters has been emerging as the most viable alternative to the conventional approaches used so far for improved metabolite production (Lv et al., 2016). Despite the abundant presence of transporters in the plants (Larsen et al., 2017a; Tang et al., 2020) (approximately 10% of the *Arabidopsis thaliana* genome encodes transporter proteins), only a handful are having their function and substrate specificity well deciphered by experimental evidence, especially the ones involved in the transport of SMs. Thus, it is challenging to find the appropriate candidates for transporter engineering to accomplish the long-term goal of systemic metabolic engineering for the betterment of plants as well as for medicinal benefits.

Considering this, better knowledge of SM transporters, particularly their diversity, functionality, regulatory parameters, approaches for their identification, and characterization is a pre-requisite for future research. In this review article, we attempt to provide a perspective and create a foundation in the field of systemic metabolic engineering by utilizing the most feasible approach of engineering the potential transporters by modulating their expression. Here, we are discussing the existing data as well as updating the understanding of plant SM transporters. Few of the previously published reviews (Shitan et al., 2014a; Lv et al., 2016; Shitan, 2016; Gani et al., 2021) have mostly dealt with various SM transporters and their potential role in metabolic engineering in plants. However, meager information is available on the diverse functionalities of transporters and the factors linked to their regulation. Herein, we extracted relevant information for more than 60 different transporters from various important plants to obtain a comprehensive picture and to develop a better understanding of plant SM transporters. The present review also reflects on the latest advancement of knowledge in the area, which could be translated for systemic metabolic engineering in plants.

## Plant Secondary Metabolite Transporters: Functional Relevance and Approaches for Their Discovery and Characterization

Plants are the producers of a significant number of specialized metabolites, which are also commonly known as SMs. SMs are small organic compounds that can be further categorized into terpenoids, alkaloids, flavonoids, and other phenolic compounds (Razdan, 2018). These metabolites not only play the role of bioactive compounds for therapeutic purposes but also naturally help combat the biotic and abiotic stress conditions by acting as defense molecules for the plants (Naik and Al-Khayri, 2016). The majority of SMs are synthesized, stored, and functionally activated at different locations in the plant system hence their mobilization from source to sink (site of synthesis to site of accumulation or activation) is necessary. In contrast to primary metabolites, the source-sink relationship is highly dynamic for the partitioning of SMs in the plants. The biosynthesis of main primary metabolites takes place exclusively in the leaves via the process of photosynthesis, therefore leaves are the major source tissue from where the one-way transport of metabolites is directed toward various sink tissues such as tuber, seed, stem, fruit, and roots (Griffiths et al., 2016). On the contrary, the biosynthesis of SM pool is not restricted to a specific tissue and many metabolites ultimately travel using various transporters from their source tissues to respective sink tissues either for accumulation or functional activation. Additionally, few specialized SMs are also involved in the transporter-mediated long-distance translocation via xylem and/or phloem pathways (Nour-Eldin and Halkier, 2013). For instance, in *Nicotiana tabacum*, the transport of nicotine from roots to leaves is mediated by the xylem pathway (Shoji et al., 2009) while in *A. thaliana* glucosinolates translocate from leaves to seeds, rosette to roots, and roots to rosette possibly via xylem and phloem mediated pathways (Andersen et al., 2013). For such types of long-distance mobilization of metabolites it may be possible that multiple transport events are needed, which might involve transporters localized at the plasma membrane and vacuolar membrane of roots and leaves (Yazaki et al., 2007). Therefore, it is believed that the transport of the SM pool is supposedly a two-way process because some metabolites travel upward while others move in the downward direction due to their diverse functional requirements at different locations in the plant. Further, various tissues can act as a source or a sink depending on the expression level of substrate biosynthetic pathway and transporter genes (Jorgensen et al., 2015). The distribution of SMs at the intercellular and intracellular level is considered as a fundamental part of growth, development, and mode of cell-environment communication (Schulz, 2011) yet, there exists a huge gap in understanding the mechanisms of transport. Plant metabolites are known to travel via several modes such as simple diffusion, membrane vesicle-mediated, and substrate-specific transporters (Shitan, 2016). Several of the plant SMs get transported by importer or exporter proteins, possibly located on the vacuolar and/or plasma membrane of the cell.

With the availability of biological resources, the development of experimental approaches, and combinatorial use of various tools (Table 1) such as bioinformatics, genomics, transcriptomics, proteomics, forward or reverse genetics, expression in the heterologous host, and transporter activity assays, it has become relatively easy to predict and identify potential transporters in the plants (Barbier-Brygoo et al., 2001; Kell et al., 2015; Lv et al., 2016; Larsen et al., 2017a; Tang et al., 2020). Many of these tools/techniques have also been repeatedly sought-after for the discovery and characterization of several plant SM transporters. Still, their functional validation and substrate specificity determination remains immensely challenging. In the recent past, genome-wide studies alone or in combination with RNA-Seq analysis followed by qRT-PCR validation have been successfully implemented to predict/identify potential transporters (Wen et al., 2020; Sun et al., 2021). In addition, co-expression analysis using known biosynthesis pathway genes also assists in identifying co-regulatory transporters (Biala et al., 2017; Larsen et al., 2017b; Yan L. et al., 2021). Further, several web servers such as WoLF PSORT, Cell-PLoc 2.0, and TMHMM have been commonly used for the prediction of subcellular localization and transmembrane topology (Wen et al., 2020; Mackon et al., 2021). For the comprehensive functional characterization of transporters, experimental validations using virus-induced gene silencing (VIGS), RNA interference (RNAi), and the study of mutant libraries have been of great use (Larsen et al., 2017a; Tang et al., 2020). However, the recently emerged genome editing tools like transcription activator-like effector nuclease (TALEN) and clustered regularly interspaced short palindrome repeats (CRISPR) have efficiently modified the transporters of various crops including *Zea mays* (Zhang et al., 2017) and *Oryza sativa* (Zhou et al., 2015; Nieves-Cordones et al., 2017) but their applicability in the study of SM transporters still awaits further research. The expression of transporters in the heterologous host systems such as mutant yeast strains and *Xenopus* oocyte cells has also been extremely valuable to study the actual functioning in the intracellular environment (Tang et al., 2020). However, these do not necessarily act as the most efficient hosts (Jørgensen et al., 2017; Dastmalchi et al., 2019) because expression levels of the transporter might get affected due to codon usage bias (Wu et al., 2021). Therefore, the *Agrobacterium*-mediated transformation of *Nicotiana benthamiana* leaves (Wu et al., 2021) and *N. tabacum* Bright Yellow 2 (BY-2) cells (Hakkinen et al., 2018) has emerged as the most convenient, compatible, and quick tool which has been successfully used to study the expression of various plant transporters (Francisco et al., 2013; Khare et al., 2017; Demurtas et al., 2019). Interestingly, a recent study has reported that yeasts can be specifically engineered with required transport machinery and co-factors to regulate the biosynthesis and accumulation of medicinal compounds (Srinivasan and Smolke, 2021).

**TABLE 1:** Potential *in silico* and experimental approaches for the identification, functional annotation, and characterization of various plant secondary metabolite transporters.

Sr. No.	Approaches	Tools and techniques	References
1.	Bioinformatics	Nucleotide and protein homology search, phylogenetic analysis, prediction of protein family, identification of conserved domains or motifs, prediction of transmembrane helices, membrane topology analysis, prediction of subcellular localization	M’mbone et al. (2018), Demurtas et al. (2019), Mackon et al. (2021)
2.	Genomics	Fully sequenced genome search, expressed sequence tag databases, study of *cis*-regulatory elements, identification of genomic clusters, plant gene predictions, plant genome annotation databases, functional libraries for plant transporter systems	Ofori et al. (2018), Wen et al. (2020), Sun et al. (2021), Yan L. et al. (2021)
3.	Transcriptomics	RNA sequencing technology, transcriptome mining, gene annotation and functional classifications, differential gene expression analysis, co-expression analysis, cDNA AFLP based transcript profiling	Zhang et al. (2013), Biala et al. (2017), Larsen et al. (2017b)
4.	Proteomics	Analysis of subcellular membrane fractions/vesicles, 2D poly acrylamide gel electrophoresis- mass spectrometry (MS), 1D SDS PAGE- MS/MS, MALDI-TOF, peptide sequences search to find homologs in various databases, function prediction on the basis of sequence-specific pattern, motif, signature/fingerprints of proteins	Barbier-Brygoo et al. (2001), Shitan et al. (2013), Wang Y. Q. et al. (2017)
5.	Genetic approaches	Forward genetics-identification of natural plant mutants for phenotypic and substrate-based variations, mutant complementation; Reverse genetics-creation of loss of function mutations by insertional mutagenesis, T-DNA elements, transposons, RNA interference, TALEN, and CRISPR/Cas9	Barbier-Brygoo et al. (2001), Larsen et al. (2017a), Payne et al. (2017), Tang et al. (2020)
6.	Heterologous host system	Gene cloning and expression in heterologous host system such as *Saccharomyces cerevisiae*, *Xenopus laevis* oocytes, mammalian cell lines, and plant-based systems (*Nicotiana tabacum* BY-2 cells and *Nicotiana benthamiana* leaves), functional complementation studies	Hakkinen et al. (2018), Srinivasan and Smolke (2021), Wu et al. (2021)
7.	Transporter activity and localization	Biochemical assays using specific substrate or inhibitor, scintillation counting of radiolabelled substrates, enzymatic assay, transportomic assay, *in-vitro* proteoliposome system, electrophysiological methods, fluorescence-based biosensors, study of GUS and GFP based fusion proteins, gene expression studies by qRT-PCR	Shitan et al. (2003), Fu et al. (2017), Behrens et al. (2019), Demurtas et al. (2019)

*AFLP, amplified fragment length polymorphism; BY, bright yellow; Cas, CRISPR associated protein; CRISPR, clustered regularly interspaced short palindrome repeats; GFP, green fluorescent protein; GUS, β-glucuronidase; MALDI TOF, matrix-assisted laser desorption ionization time of flight; MS, mass spectrometry; TALEN, transcription activator like effector nuclease.*

**FIGURE 1:**
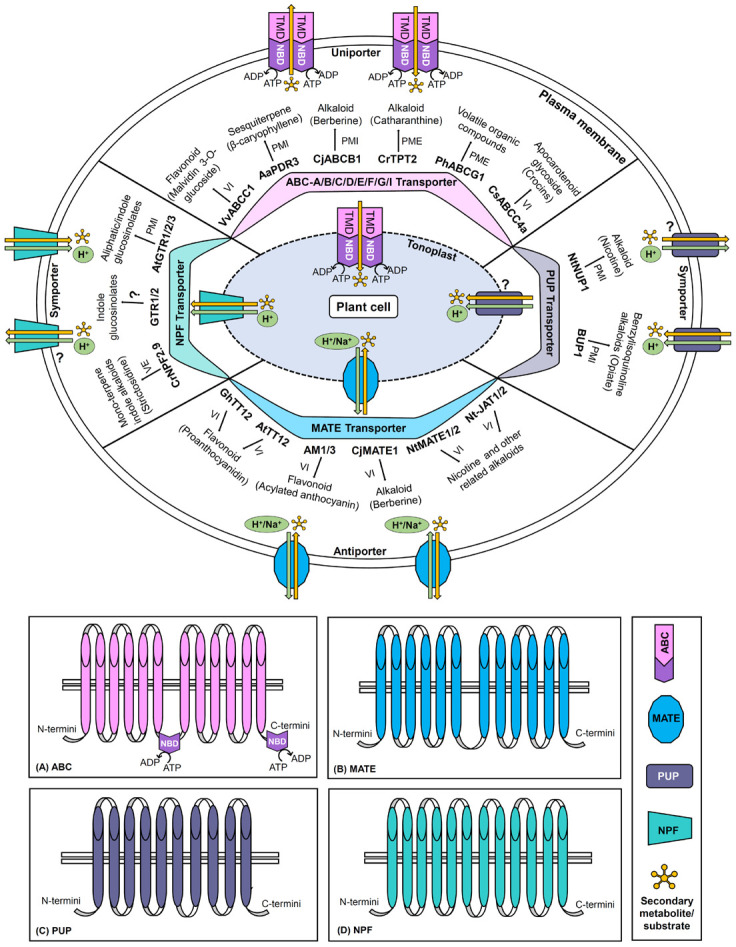
Schematic representation of ABC, MATE, PUP, and NPF transporters. The diagram denotes transporter classification, localization (plasma membrane/tonoplast), general structure, directionality, energy source (ATP hydrolysis/proton or other cation gradients), domains and subfamilies (only for ABC transporter), and transport mechanisms (uniport/symport/antiport). For each category of the transporter family, important examples of plant secondary metabolite transporters along with their corresponding target metabolites are illustrated. The panels **(A–D)** represent the generalized topology of the transporters belonging to the ABC, MATE, PUP, and NPF families, respectively. ABC, ATP binding cassette; MATE, multidrug and toxic compound extrusion; NPF, nitrate and peptide transporter family; PME, plasma membrane exporter; PMI, plasma membrane importer; PUP, purine uptake permease; VE, vacuolar exporter; VI, vacuolar importer (? represents that enough information is not available).

The present review provides the current perspective of a large number of SM transporters particularly discovered in the past two decades from various important plant species (Supplementary Table 1). Herein, the classification, structural, mechanistic, and functional aspects of four major transporter families namely ATP Binding Cassette (ABC), multidrug and toxic compound extrusion (MATE), purine uptake permease (PUP), and nitrate and peptide transporter family (NPF) have been discussed to better comprehend their role in the transport of specific plant SMs (Table 2; Figure 1).

**TABLE 2:** A brief comparison of structural and functional features of various transporter families.

Transporter family	No. of trans-membrane helices	Source of energy for transport	Nature of transporter	Conserved sequence	Major secondary metabolite substrates	Subunits	References
ABC	∼12	ATP hydrolysis	Uniporter	Highly conserved walker A (GXXGXGKS/T), walker B (JJJJDE) and signature (LSGGQ) motifs are present in the NBD domain (play role in ATP binding and hydrolysis)	Alkaloids, terpenoids, flavonoids and other phenolics, VOCs, and apocarotenoids	Multi subunits (NBD and TMD)	Kang et al. (2011), Bailly (2014), Hwang et al. (2016)
MATE	∼12	Cation (H^+^/Na^+^) gradient	Cation antiporter	No legitimate stretch of conserved amino acids is present but in few cases the C-lobe of the protein possesses glutamic acid and aspartic acid to form a conserved pocket (probably proton binding pocket)	Alkaloids and flavonoids	Single subunit	Kusakizako et al. (2019), Upadhyay et al. (2019)
PUP	∼9–10	Proton (H^+^) gradient	Proton symporter	No universally conserved region is reported but MSA of several PUPs revealed the presence of various conserved amino acids	Nicotine and few other alkaloids	Single subunit	Jelesko (2012), Kato et al. (2015), Dastmalchi et al. (2019)
NPF	∼12	Proton (H^+^) gradient	Proton symporter	No stringently conserved motif is present, but in several cases, TMH1 contain ExxER/K conserved motif (proton binding sequences) while few others have ExxDR/K or DxxDR/K as a conserved motif	Glucosinolates and alkaloid derivatives	Single subunit	Sun et al. (2014), Corratge-Faillie and Lacombe (2017), Longo et al. (2018)

*ABC, ATP binding cassette; MATE, multidrug and toxic compound extrusion; MSA, multiple sequence alignment; NBD, nucleotide binding domain; NPF, nitrate/peptide transporter family; PUP, purine uptake permease; TMD, transmembrane domain; TMH, transmembrane helix; VOC, volatile organic compounds. X = any amino acid, J = aliphatic amino acid.*

### ATP Binding Cassette Transporters

ATP Binding Cassette proteins are ubiquitously found in all organisms including plants, animals, and fungi, amongst which the majority are known to act as transporters. ABC proteins represent one of the largest families of transporters in plants and are involved in a plethora of biological functions such as growth, development, reproduction, detoxification, stomatal regulation, phytohormone transport, nutrient uptake from the soil, pathogen defense, fungal symbiosis, transport of primary and SMs, and so on (Kang et al., 2011; Shoji, 2014; Borghi et al., 2019; Banasiak et al., 2021; Do et al., 2021). So far, 154 putative ABC members have been identified in *Solanum lycopersicum* (Ofori et al., 2018) followed by more than 120 members each in *A. thaliana* (Sanchez-Fernandez et al., 2001), *Z. mays* (Pang et al., 2013), *O. sativa* (Garcia et al., 2004), and grapevine (*Vitis vinifera*) (Cakir and Kilickaya, 2013). Besides, strawberry (Shi et al., 2020), pineapple (Chen et al., 2017), *Lotus japonicus* (Sugiyama et al., 2006), and rubber tree (*Hevea brasiliensis*) (Zhiyi et al., 2015) have also been known to possess 115, 100, 91, and 46 genes, respectively, which encode for putative ABC transporters. Generally, ABC transporters possesses two types of domains, namely cytosolic nucleotide-binding domain (NBD)/ATP binding domain and transmembrane domain (TMD) (Sharom et al., 2011). A typical functional ABC transporter harbors two NBDs and two TMDs (Figure 1) and if these four domains are present in a single polypeptide chain, it is termed a full-size transporter. Whereas if only one NBD and one TMD are present in a single polypeptide chain, it is termed half-size transporter that needs to form a homo- or heterodimer to act as a functional unit. Based on the combination and number of TMDs/NBDs, the ABC transporter family has often been grouped into nine subfamilies viz. ABCA, ABCB, ABCC, ABCD, ABCE, ABCF, ABCG, ABCH, and ABCI (Verrier et al., 2008) amongst which ABCH has not been identified in plants. Besides, a recent study has proposed that considering the highly conserved nature of NBDs, the structural homology of TMDs should be the only basis for the categorization of ABC transporters (Thomas et al., 2020). Although, the crystal structure is not yet available for the plant ABCs but the structural information of other ABC transporters indicates the involvement of alternating access mechanisms for the transport of various types of substrates (Locher, 2016). The alternation of inward and outward facing conformational structures of the transporter is believed to regulate the substrate binding or release (Beis, 2015; Yu et al., 2021). The cryo-EM studies have also successfully identified the bound substrate in the inward-facing conformation of a few eukaryotic ABCs (Januliene and Moeller, 2020).

The prevalence of ABC members has been well documented in the transport of a diverse range of plant SMs including alkaloid, terpenoid, flavonoid, volatile compounds, and apocarotenoid. The function of alkaloid-specific ABC transporters has been studied in two important medicinal plants *Coptis japonica* (Yazaki et al., 2001; Shitan et al., 2013) and *Catharanthus roseus* (Yu and De Luca, 2013). In *C. japonica*, a medicinally valued benzylisoquinoline alkaloid (BIA) namely berberine is produced, which is known for its antimicrobial properties. Berberine is synthesized in the roots and possibly further accumulates into the rhizome of the plant. Two transporters from the ABCB subfamily i.e., CjABCB1 and CjABCB2 have been characterized and found to be involved in the influx of berberine into the rhizome (Yazaki et al., 2001; Shitan et al., 2013). Further, northern hybridization deduced a positive correlation between berberine accumulation and the predominant expression of *CjABCB1* in the rhizome (Yazaki et al., 2001). Similarly, time-dependent uptake by CjABCB1 showed a higher accumulation of berberine in the *Xenopus* oocytes. Whereas the treatment with ABC transporter inhibitors nifedipine, verapamil, and glibenclamide has significantly reduced the berberine uptake, which demonstrates that CjABCB1 certainly mediates the transport of berberine. The drug sensitivity assays performed in *Saccharomyces cerevisiae* AD1–8 demonstrated that in addition to the berberine, CjABCB1 is also capable of recognizing other compounds such as 4-nitroquinoline N-oxide and reticuline (Shitan et al., 2003). Likewise, the role of CjABCB2 was primarily determined based on sequence similarity and phylogenetic relationship with CjABCB1. Further, functional studies have also revealed that CjABCB2 is closely related to CjABCB1 and both the transporters are localized on the plasma membrane of the rhizome and translocate berberine from roots to the rhizome, possibly via xylem mediated transport (Shitan et al., 2013). Similarly, Yu and De Luca (2013) have also identified the alkaloid-specific transporter CrTPT2 in *C. roseus* belonging to pleiotropic drug resistance (PDR) transporters. PDR is part of the ABCG subfamily that particularly has an arrangement of NBDs and TMDs in reverse orientation (Lv et al., 2016). Dimeric monoterpene indole alkaloid (MIA) compounds vinblastine and vincristine have been reported to produce in *C. roseus*, which possess anti-carcinogenic properties. The process of their dimerization involves the coupling of monomeric precursor units namely catharanthine and vindoline. Catharanthine biosynthesis occurs in the leaf epidermis while its accumulation has entirely been observed on the leaf surface, which indicates the potential role of CrTPT2 in the efflux of catharanthine. Further, VIGS mediated inactivation of *CrTPT2* leads to enhanced accumulation of catharanthine within the leaf epidermis while 30–50% reduction was observed onto the leaf surface, which validated that CrTPT2 is a catharanthine specific transporter. Similarly, substrate specificity assays have also confirmed that CrTPT2 is highly specific for catharanthine compared to other MIAs (Yu and De Luca, 2013). Additionally, ABC transporters have also been predicted in *Capsicum* species which are usually considered as a horticultural crop for dietary purposes but the *Capsicum* also contains capsaicinoid compounds, namely capsaicin and dihydrocapsaicin, that provide health-promoting benefits (Qin et al., 2014). Capsaicinoids are SMs of the alkaloid category, which possess medicinal value due to their anti-bacterial, anti-carcinogenic, and antioxidant properties (Perla et al., 2018). RNA-Seq data (Kim et al., 2014) and qRT-PCR analysis highlight the presence of putative ABCC and ABCG members, which might be involved in the transport of capsaicin and dihydrocapsaicin (Lopez-Ortiz et al., 2019).

Furthermore, ABC-mediated transport of terpenoids has been studied in the Chinese medicinal plant *Artemisia annua* L. which produces a sesquiterpenoid named β-caryophyllene that is known to have antioxidant, antibiotic, anti-inflammatory, and anti-carcinogenic activities (Fu et al., 2017). *A. annua* contains two types of trichomes namely glandular and T-shaped trichomes which are the main sites for SM production. The detailed functional studies suggest that glandular trichomes possess a biosynthetic pathway for artemisinin which is used as an anti-malarial drug, while the information for T-shaped trichomes remains largely undiscovered (Zhang et al., 2012; Soetaert et al., 2013). Interestingly, the RNA-Seq analysis of the T-shaped trichome has predicted a potential transporter AaPDR3 which is localized on the plasma membrane and is probably linked with the transport and biosynthesis of β-caryophyllene (Fu et al., 2017). The content of β-caryophyllene was found to be increased upon overexpressing *AaPDR3* while its reduced accumulation was observed by RNAi-induced downregulation of the transporter. Further, the heterologous expression has also demonstrated that AaPDR3 specifically transports β-caryophyllene. Fu et al. (2017) also indicated that AaPDR3 seems to have a positive effect on the biosynthesis of β-caryophyllene by preventing its accumulation at the source tissue and thus, avoiding the negative feedback inhibition. Moreover, terpenoid transporters have also been identified in the tobacco species that are known to produce a diterpene sclareol in response to pathogen attacks. For example, NpPDR1 (Stukkens et al., 2005) and NtPDR1 (Pierman et al., 2017) are plasma membrane-localized transporters from *Nicotiana plumbaginifolia* and *N. tabacum*, respectively. These transporters are involved in the secretion of sclareol to the leaf surface, which is the major antifungal defense compound of the *Nicotiana* genus. Similarly, NtPDR1 (Pierman et al., 2017) and Nb-ABCG1/2 (Shibata et al., 2016) transporters from *N. tabacum* and *N. benthamiana*, respectively are also involved in the secretion of antimicrobial sesquiterpenoid named capsidiol. In addition, ABC members also assist in the transport of specific flavonoids, as reported for *V. vinifera* whose exocarp significantly accumulates anthocyanins, which are synthesized in the cytosol and supposedly get stored in the vacuole. The phylogenetic analysis (Francisco et al., 2013) of various ABC proteins from *V. vinifera* indicated that VvABCC1 has shown high sequence similarity with *Z. mays* ZmMRP3 transporter (Goodman et al., 2004), which is reported to participate in the vacuolar transport of anthocyanin. Further, *VvABCC1* was observed to be highly expressed in the exocarp and the functional studies also confirmed its potential role in the transport of anthocyanin from cytosol to vacuolar lumen of the exocarp. Moreover, the uptake assays have revealed that VvABCC1 is specific only toward anthocyanins as the other flavonoid from non-anthocyanin categories such as epicatechin did not show any transport (Francisco et al., 2013).

The function of ABC transporters is also implicated in the emission of volatile organic compounds (VOC) from the plant. General assumption dictates that VOCs may simply emit out from their biosynthetic site such as flowers via the process of diffusion; but being a slow process, diffusion can create the temporary accumulation of VOCs at the membranes, which might be toxic for the integrity and functioning of the cellular membranes. Thus, active transport has also been predicted for the emission of VOCs. To identify the putative VOC transporter, *Petunia hybrida* has been chosen, which produces phenylpropanoid and benzenoid as volatiles. Examining the RNA-Seq datasets of *P. hybrida* revealed the presence of ABC transporter from the G subfamily named PhABCG1, which is predicted to be located on the plasma membrane and possibly also related to the biosynthesis of VOCs. The RNAi-mediated knockdown of *PhABCG1* reduces more than 50% emissions of VOCs and at the same time, their corresponding internal amount is also enhanced. The study certainly indicates the potential role of PhABCG1 in the active transport of volatiles emitted from flowers of *P. hybrida* (Adebesin et al., 2017).

Apart from VOCs, the transport of apocarotenoids has also been reported in the *Crocus sativus* (saffron), which is one of the most expensive and valuable spices in the world. The red appearance of saffron arises due to the presence of glycosylated apocarotenoids commonly known as crocins, which are probably synthesized in the cytosol and further transported to vacuolar lumen for storage. Since saffron is obtained from the dried stigma of *C. sativus*, its transcriptome was searched for putative transporters. The analysis identified CsABCC4a protein which is expressing specifically in the stigma and might be employed for the transport of crocins to the vacuole. Further, the co-expression studies of *CsABCC4a* with crocin biosynthetic pathway enzymes also showed a positive correlation. Finally, the localization and functional studies have validated the potential role of CsABCC4a in the vacuolar accumulation of crocins (Demurtas et al., 2019).

### Multidrug and Toxic Compound Extrusion Transporters

Multidrug and toxic compound extrusion transporter family is found in all possible living organisms with varied substrate specificities. Amongst all prokaryotes and eukaryotes, plants have been comprised of a relatively abundant range of MATE members (Santos et al., 2017). MATE family exhibit a wide array of functions in plants including transport of SMs and phytohormones, detoxification of xenobiotics, aluminum tolerance, ion homeostasis, organ differentiation, disease resistance, and so on (Shoji, 2014; Upadhyay et al., 2019). To date, 56 MATE members have been reported from *A. thaliana* (Wang et al., 2016), 117 from *Glycine max* (Liu et al., 2016), 45 from *O. sativa* (Wang et al., 2016), and 70 from *Medicago truncatula* (Wang J. et al., 2017). Bioinformatic studies conducted in several plants viz. *S. lycopersicum* (Santos et al., 2017), *Raphanus sativus* (M’mbone et al., 2018), and *Coptis deltoidea* (Zhong et al., 2020) have also predicted or in some cases identified several MATE family members having a potential role in the transport of SMs. Although, a prominent fraction of MATE members function as the exporters by antiport mechanism using an electrochemical gradient generated by H^+^ or Na^+^ cations (Figure 1). However, the tonoplast localized MATE members can also act as influx transporters for vacuolar sequestration of metabolites (Liu et al., 2016; Behrens et al., 2019). Typical MATE transporters have a unique topology of twelve transmembrane helices arranged in the form of intramolecular two-fold symmetry (Kusakizako et al., 2019; Upadhyay et al., 2020). Due to vast substrate diversity, this family does not possess any legitimate stretch of conserved amino acids in the core domain structure. However, the C-lobe of MATE protein is believed to contain certain conserved acidic residues (Kusakizako et al., 2019). Owing to the crystallographic structures and atomic simulation methods, the transport mechanisms have been well studied for various prokaryotic and eukaryotic MATE members (Upadhyay et al., 2019). Primarily the rocker-switcher mechanism is reported for plant MATE transporters which involves two interconverting conformational states of the transporter i.e., straight and bent. The transition between the two states having differential binding affinities for the substrate depends on the protonation of conserved acidic residues and the rearrangements of transmembrane helices. However, a recent study has also proposed alternating access mechanism of transport by MATE transporters which shows that isomerization among different conformational states of the transporter controls the binding of the substrate (Claxton et al., 2021).

The MATE family members are reported to participate in the transport and accumulation of various plant SMs from the alkaloids and flavonoids categories. For instance, *N. tabacum* biosynthesizes a specific alkaloid in its roots commonly known as nicotine whose translocation to the other plant parts is believed to be mediated by MATE members. The close phylogenetic relationship with *A. thaliana* TT12 protein, which is a potential transporter for the vacuolar import of proanthocyanidin precursors led to the identification of NtMATE1 and NtMATE2 transporters in *N. tabacum* (Shoji et al., 2009). These transporters are proposed to be involved in the sequestration of nicotine and other related alkaloids in the root vacuole. The methyl jasmonate (MeJ) induced upregulation of *NtMATE1/2* was found to be in coherence with the nicotine biosynthetic pathway gene such as putrescine N-methyltransferase, which indicate their participation in the transport of nicotine. The MeJ elicitation also decreases the cytoplasmic pH of the root cells that is probably due to the import of protons from the vacuole, which further demonstrates that NtMATE1 utilizes the proton gradient in exchange for the vacuolar uptake of nicotine. The *in vitro* substrate uptake assays have revealed that in addition to nicotine, the NtMATE1 is also capable of transporting tropane alkaloids such as scopolamine and hyoscyamine with lower affinities (Shoji et al., 2009). Similarly, another vacuolar importer for alkaloids was also reported in *N. tabacum* after the functional characterization of a novel transporter Nt-JAT1 (Morita et al., 2009). It was reported to localize on the tonoplast of leaf cells and is likely to be involved in the xylem mediated transport of alkaloids from roots to the vacuolar lumen of the leaf, which is considered as their major accumulation site. The root synthesized nicotine further needs to be translocated to the aerial parts of plants to act as a defensive toxin against herbivores and pathogens (Shitan et al., 2009). Besides, it has also been observed that nicotine is not the exclusive substrate for Nt-JAT1, in fact, it also recognizes other alkaloids such as anabasine which is the endogenous alkaloid of tobacco. Additionally, hyoscyamine and berberine, which belong to other plant species can also be transported if provided exogenously (Morita et al., 2009). A closely related Nt-JAT2 transporter was also discovered in *N. tabacum* (Shitan et al., 2014b), which perhaps acts in conjunction with Nt-JAT1 and performs a similar function for nicotine accumulation in the vacuolar lumen of leaf tissues. Primarily to combat herbivory attack Nt-JAT2 specifically accumulates nicotine and other alkaloids in the leaves while Nt-JAT1 majorly helps maintain a steady state of alkaloid distribution (Shitan et al., 2014b). Furthermore, another important alkaloid called berberine from a medicinal plant *C. japonica* is also reported to be translocated by a MATE transporter that perhaps functions along with previously discussed (section “ATP Binding Cassette Transporters”) ABC transporters (CjABCB1/2) which suggested that berberine is synthesized in the roots and further transported to the sink tissue rhizome (Shitan et al., 2013). However, the underlying mechanism for further accumulation of berberine in the rhizome was not clear until the discovery of CjMATE1. The study of membrane vesicles from cultured *C. japonica* cells indicated transport of berberine by a proton antiporter which was identified from expressed sequence tag library and designated as CjMATE1 transporter. The berberine accumulation in cultured cells was positively coordinating with the expression levels of *CjMATE1* and GFP fusions have also revealed its vacuolar localization, which confirmed that CjMATE1 certainly participates in vacuolar sequestration of berberine. Altogether, these findings imply that more than one transporter is involved in the translocation and accumulation of berberine from source to sink tissue (Takanashi et al., 2017).

Apart from alkaloids, the translocation of various phenolic compounds particularly from flavonoid families such as proanthocyanidins (PA) and anthocyanins are also mediated by MATE transporters. *V. vinifera* has been identified with two groups of transporters namely anthoMATEs (AM1, AM3) (Gomez et al., 2009) and putative MATEs (VvMATE1 and VvMATE2) (Perez-Diaz et al., 2014), which are involved in the transport of acylated anthocyanins and PAs respectively. Amongst these, functional studies have only been performed for the former group which showed that expression of *AM1* and *AM3* is majorly confined to berry skin, that is the main site for anthocyanin biosynthesis. Except for VvMATE2, which is predicted to be localized on the plasma membrane, the remaining three MATE transporters are located onto the tonoplast. Taken together, these studies corroborate that AM1 and AM3 are probably involved in vacuolar sequestration of acylated anthocyanins (Gomez et al., 2009). However, the exact mechanism has not yet been demonstrated for VvMATE1/2, but probably these are also involved in the vacuolar accumulation of PAs (Perez-Diaz et al., 2014). Another PA transporter from the MATE family is reported from *Gossypium hirsutum* L. (commonly known as upland cotton), which is a highly valuable widely grown commercial crop. This variety naturally produces brown fibers, thus it is also named brown cotton, and it has been reported that PA is the major substance behind its brown coloration. The naturally occurring brown color benefits the textile industry by reducing the dyeing and processing cost (Xiao et al., 2014). Based on the analogy with AtTT12 transporter, the GhTT12 transporter was identified from *G. hirsutum*, which is proposed to be involved in the PA translocation from cytoplasm to the vacuole, and the vacuolar localization is further confirmed by GFP fusion studies (Gao et al., 2016). In addition to the brown cotton, the relative expression of GhTT12 was also determined in low PA-containing white cotton, and the comparative analysis demonstrated higher expression of transporter in the former case, which ascertains the role of GhTT12 as potential PA transporter (Gao et al., 2016). Similarly, phylogenetic analysis and qRT-PCR expression studies have identified three more potential transporter candidates namely GhMATE12, GhMATE16, and GhMATE38 from *G. hirsutum* (Xu et al., 2019), which might be involved in the transport and accumulation of proanthocyanidins probably via the same mechanism employed by the GhTT12 transporter.

### Purine Uptake Permease Transporters

The PUP family is rather an emerging class of plant transporters that was primarily studied in *A. thaliana* and reported to be involved in the transport of adenine, cytosine, and purine derivatives (Gillissen et al., 2000). Moreover, the presence of PUP related genes has also been revealed in many plants such as *O. sativa* (Qi and Xiong, 2013), *S. lycopersicum*, *N. tabacum* (Hildreth et al., 2011), *Papaver somniferum* (Dastmalchi et al., 2019), and *Coffea canephora* (Kakegawa et al., 2019). The exact transport mechanisms are poorly understood but the PUP transporters are presumed to act as proton symporters probably by using secondary active transport mechanisms (Figure 1; Jelesko, 2012). Although the detailed characterization of the PUP family is yet to be exhibited, still the limited information available on transporter topology has been predicted using THMM1.0, which revealed the presence of 9–10 transmembrane helices in a large gene family of purine permeases from *A. thaliana* (AtPUP1–AtPUP15). Further, the substrate specificity of AtPUP1 was determined by functional complementation assays in a yeast mutant, which has shown that AtPUP1 is a high-affinity transporter for adenine and cytosine (Gillissen et al., 2000). Similarly, another study (Zurcher et al., 2016) has investigated that PUP14 of *A. thaliana* is likely to be involved in plant morphogenesis by regulating the transport and distribution of the cytokinin pool. Interestingly, in addition to the previously mentioned substrates, PUP members have expanded their uptake activity with non-purine derivatives such as vitamin B6 and plant SMs, particularly nicotine and few other related alkaloids (Kato et al., 2015).

The functional role of PUP homologs in alkaloid transport was first discovered and characterized in *N. tabacum* containing a specific transporter for the uptake of nicotine which is known as Nicotine Uptake Permease-1 (NUP) (Hildreth et al., 2011). In tobacco, nicotine biosynthesis is restricted to root cells, particularly the root tips, from where the major portion mobilizes to the above-ground plant parts while some amount reaches the rhizosphere which can further be taken back into the roots by NUP1 mediated uptake (Hildreth et al., 2011; Kato et al., 2015). Therefore, the maximum transcript of *NUP1* was obtained in the roots and as demonstrated by GFP fusions subcellular localization was confirmed at the plasma membrane. Further, the *NUP1* knockdown mediated reduced nicotine accumulation in the hairy root cells also confirmed substrate specificity which was also evident by nicotine uptake assays in *Schizosaccharomyces pombe*. Altogether, these findings suggest that NUP1 is perhaps involved in the movement of nicotine from apoplast to cytosol of root cells (Hildreth et al., 2011; Jelesko, 2012) and probably functions in coordination with other transporters such as NtMATE1/2 and Nt-JAT1/2, for maintaining the nicotine homeostasis between root and shoot (Shitan et al., 2015). Similarly, a recent report (Dastmalchi et al., 2019) has also identified PUP-like transporters in a medicinal plant *P. somniferum* (commonly known as opium poppy), which mediate the transport of various opiates belonging to a diverse class of BIA. *P. somniferum* is the prime natural source of BIAs and several therapeutic compounds or their precursors such as morphine, codeine, and thebaine (Knutsen et al., 2018). The genome sequence of *P. somniferum* revealed the presence of PUP-like genes which presumably function as BIA transporters. The distinct set of six PUP homolog genes were named benzylisoquinoline alkaloid uptake permeases (BUP) 1–6, due to their clustering with BIA biosynthetic pathway genes (Dastmalchi et al., 2019). The opiate biosynthesis mainly occurs in sieve elements followed by the movement of various opiate intermediates to the laticifers cells for later steps to be carried out. In this context, the BUP1 was elucidated as an opiate importer located on the plasma membrane of laticifers cells. Further, functional studies have demonstrated that BUP1 is probably involved in the transport of opiate alkaloids from sieve elements to laticifers cells apparently via the apoplastic route (Dastmalchi et al., 2019).

### Nitrate and Peptide Transporter Family Transporters

The nitrate and peptide transporter family, which is commonly abbreviated as NRT/PTR or NPF has supposedly been present in all organisms and is known to act as proton symporters (Figure 1; Corratge-Faillie and Lacombe, 2017). Until recently, plant NPF members were thought to be transporting only nitrate and peptide (Hammes et al., 2010; Qiu et al., 2017). However, unlike their bacterial and animal counterparts, plant NPF transporters are quite heterogeneous (Nino-Gonzalez et al., 2019) and comparatively very high in number with an extended range of substrate specificities such as nitrate, dipeptide, phytohormones [indole acetic acid (IAA), abscisic acid (ABA), jasmonic acid (JA), and gibberellic acid (GA)], amino acids, chloride, and plant SMs (Corratge-Faillie and Lacombe, 2017). Whereas, some of the NPF members are also reported to exhibit dual substrate specificities (Sun and Zheng, 2015). The genome-wide sequence analysis (Longo et al., 2018) has revealed the distribution of NPF transporters in a large number of monocots, dicots, aquatic and land plants including *O. sativa*, *Z. mays*, *Sorghum bicolor*, *S. lycopersicum*, *Carica papaya*, *L. japonicus*, etc. The most studied plant, *A. thaliana* was found to have 50 NPFs, followed by 20 and 21 in *Physcomitrella patens* and *Marchantia polymorpha*, respectively. On the other hand, a very high number has been reported in *M. truncatula* and *G. max* i.e., 92 and 114, respectively (Longo et al., 2018). The topology prediction of NPF transporters in *A. thaliana* has deciphered the presence of twelve transmembrane helices (Sun et al., 2014). Further, the potential mechanism of transport has been studied in the NPF6.3 of *A. thaliana*, which is, so far, the most well-studied transporter from the NPF family. As indicated by structural models, the substrate transport perhaps occurs via alternating access mechanism, which involves conformational changes amongst inward and outward-facing stages of the transporter. The process of transport is initiated by protonation of the specific histidine residue residing in the substrate-binding pocket of the transporter that is followed by interaction and binding of the substrate (Parker and Newstead, 2014). However, another theory suggests that while the transporter is facing inwardly an additional proton binding occurs at the conserved ExxER motif that creates an outward-facing conformation leading to binding of the substrate, which then again initiates an inward-facing arrangement required for the substrate release inside the cell (Wen and Kaiser, 2018).

The transport of plant SMs by the NPF members have been mainly studied for glucosinolates (GSLs) and MIAs. Glucosinolates are a specific class of plant SMs derived from amino acids and many of the GSLs have high nutritional value due to their rich nitrogen and sulfur content (Martinez-Ballesta et al., 2013; Jorgensen et al., 2015). GSLs are well-versed components of the plant defense system and their hydrolyzed product is also known to possess anti-carcinogenic properties. The occurrence of GSLs is attributed explicitly to the cruciferous plants such as members of the Brassicaceae family (Sonderby et al., 2010). Further, it has been speculated that GSLs are synthesized in various plant parts and eventually get distributed to different locations by GSL transporter proteins (GTRs). AtGTR1/2/3 transporters of *A. thaliana* (Nour-Eldin et al., 2012) are reported to be possibly involved in the import or export of aliphatic and indole glucosinolates depending on their localization at source/sink tissues (Jørgensen et al., 2017). The function of GTRs has also been proposed in Chinese kale (*Brassica oleracea*), which is known to be a native vegetable of china which has high nutritional value along with anti-carcinogenic and antioxidant properties due to the presence of various glucosinolates, carotenoids, and phenolic compounds (Wang Y. Q. et al., 2017). In *B. oleracea* var. *chinensis* Lei, two GSL transporters BocGTR1a and BocGTR1c were identified, which express predominantly in the leaves and buds (Jiang et al., 2019) with a variable expression pattern that also depends on the plant developmental stages and various abiotic and biotic stress conditions. In addition to similar transmembrane topology, three-dimensional structure, and functional domains, these two transporters also showed more than 81% amino acid sequence homology with *A. thaliana* GTR proteins, which indicates their related functionalities. Further, the RNAi mediated inactivation has proposed the potential role of these transporters in the translocation of root synthesized GSLs to leaves and stems. However, the exact transportation mechanism still needs more experimental validation (Jiang et al., 2019). Similarly, in the recent past, the probable role of GTRs in the allocation of GSLs has also been deciphered in response to root herbivory in *Brassica rapa*, which suggested the involvement of two transporter genes namely GTR1 and GTR2 (Touw et al., 2020). The indole and benzyl GSLs were found to be highly accumulated in the taproot of *B. rapa* during the attack of *Delia radicum*, which is possibly due to their increased biosynthesis and/or transport from distal organs. At the same time, GSL transporter and biosynthesis genes were also upregulated, which implies their role in the transport of GSLs from distal organs and/or enhanced biosynthesis in the taproot. Despite the increased expression of *GTR* genes in taproot, there was no reduction in the GSL concentration in distal tissues such as shoot but authors still hypothesized that these transporters are somehow involved in the retention of GSLs in taproot to fulfill the immediate requirement for combating the pathogen attack, although the precise molecular mechanism remained unclear (Touw et al., 2020).

Further, Payne et al. (2017) have also identified an NPF transporter CrNPF2.9 from a medicinal plant *C. roseus* that specifically transports a metabolite strictosidine which belongs to the class of MIA. Strictosidine is an intermediate metabolite in the MIA biosynthetic pathway and it has been hypothesized from transcriptomic data of *C. roseus* that CrNPF2.9 transporter is co-regulated with early MIA pathway genes, which perhaps, hints toward its involvement in the transport of MIA pathway intermediates (Verma et al., 2014). The accumulation of strictosidine in the leaf vacuole was significantly increased upon VIGS mediated suppression of *CrNPF2.9* transporter along with a sharp reduction in MIA pathway end products in the cytosol. This demonstrated that CrNPF2.9 might export strictosidine from the vacuolar lumen to the cytosol. In addition to the previously described plasma membrane localized NPF members, the discovery of CrNPF2.9 that is located onto tonoplast has corroborated the NPF mediated intracellular mobilization of metabolites (Payne et al., 2017).

## Factors Regulating the Transporter Activity and Function

**FIGURE 2:**
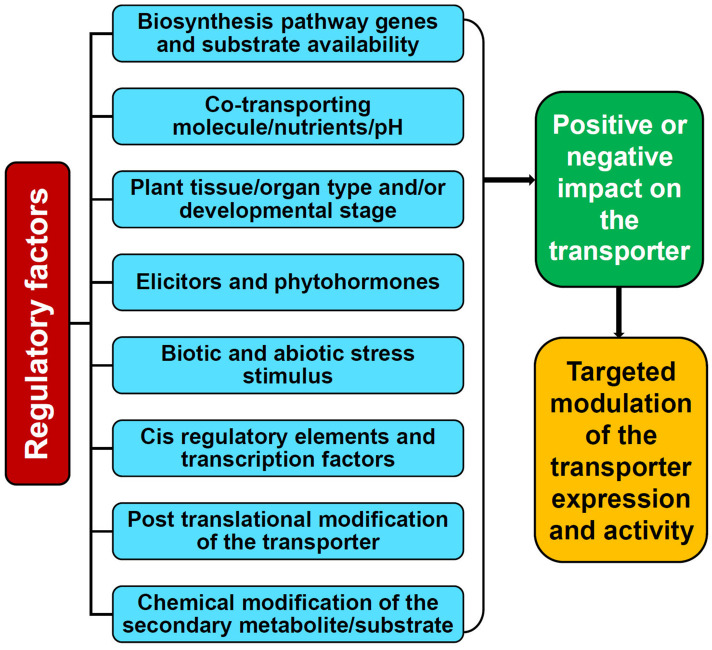
Factors affecting the activity and function of the plant secondary metabolite transporters.

**FIGURE 3:**
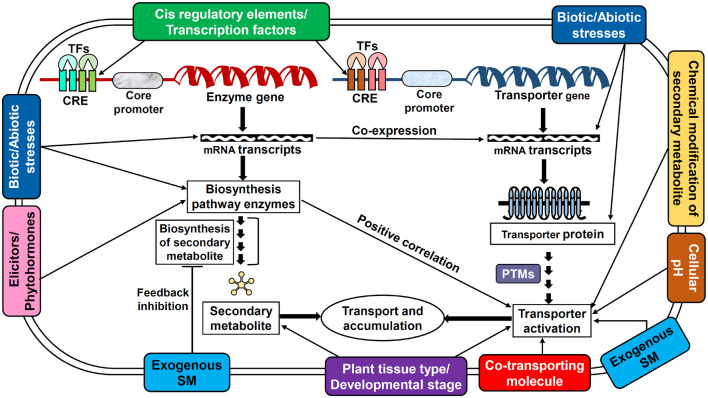
Potential regulatory mechanisms employed by several key factors affecting the transporter functioning in plants. This model displays the interaction of various regulatory factors with secondary metabolite biosynthesis and transporter protein expression/activation. The interactions also depict the possible cross-talks between metabolite biosynthesis and transporter expression. CRE, *cis*-regulatory elements; PTM, post-translational modifications; SM, secondary metabolite; TF, transcription factors.

The availability of a specific substrate in the required quantity is of the prime consideration for a transporter to be fully functional. However, the activity of transporters is not entirely dependent just on the substrate but also distinctly coordinated and regulated by several elements such as cellular and external environmental conditions, plant growth/developmental stages, nutrients, stress stimulus, tissue/organ specificity, phytohormones, elicitors, ionic gradients, etc. which are controlled at multiple levels (Figures 2, 3; Tang et al., 2020; Dhara and Raichaudhuri, 2021). Perhaps, the complexity of transport regulation also arises due to cross-talks with other metabolic pathways (Lynch et al., 2021) and multifold control points at the transcriptional, post-transcriptional, translational, and post-translational levels (Barbier-Brygoo et al., 2001).

In order to achieve the elevated availability of the substrate, the upregulation of biosynthetic pathway genes is one of the ways which can probably induce the respective transporters in a substrate-dependent manner. Thus, keen attention has been paid to perform co-expression studies between various transporters such as NtMATE1, NtMATE2 (Shoji et al., 2009), Nt-JAT1 (Morita et al., 2009), SmABCC1, SmABCG46, SmABCG40, SmABCG4 (Yan L. et al., 2021), and crucial genes from biosynthesis pathways of their substrates which have consistently shown positive co-regulatory mechanisms. In addition to altering the substrate biosynthesis pathway, the exogenous supply of substrate or substrate analogs also considerably affects the activity of transporters. For instance, the addition of sclareol to the suspension cultures of *N. plumbaginifolia* induces the expression of *NpPDR1* transporter, which exports sclareol to the leaf surface and a comparable outcome was also obtained when suspension cells were treated with sclareolide, a structural analog of sclareol (Jasiński et al., 2001; Grec et al., 2003). Similarly, the exogenous treatment of medicarpin precursors namely 4-coumarate and liquiritigenin to the roots of *M. truncatula* seedlings significantly increases the mRNA accumulation of MtABCG10 transporter (Biala et al., 2017).

Additionally, the dependency of co-transport compounds such as glutathione has also been reported for the transport of several flavonoids including malvidin 3-O-glucoside and cyanidin 3-O-glucoside by VvABCC1 (Francisco et al., 2013) and AtABCC2 (Behrens et al., 2019) transporters, respectively. On the other hand, nutrients are also found to affect the functioning of various transporters, and ABCG37 of *A. thaliana*, which transports scopoletin is one such example that gets highly activated when the plant is grown under iron-deficient conditions. The Fe deficiency probably promotes the synthesis of phenolic compounds including scopoletin, which is possibly due to the upregulation of some of its biosynthetic enzymes from the phenylpropanoid pathway that eventually led to an increase in the mRNA transcript of the transporter (Fourcroy et al., 2014). Cellular pH also control the functioning of those transporters, which utilize proton gradient as the energy source such as NtMATE1/2 significantly get upregulated upon acidification of cytoplasm (Shoji et al., 2009). Further, expression of the SM transporters is mostly restricted to the specific plant tissues and/or developmental stages owing to their functional relevance. The AaPDR3 transporter of *A. annua* which specifically expresses in T-shaped trichomes of old leaves and roots represents tissue-specific expression (Fu et al., 2017). In addition, AaPDR3 expression was also found to be developmentally regulated with a concomitant increase with that of the leaf age. Whereas, the CrTPT2 transporter of *C. roseus* is negatively regulated with leaf age and therefore the expression is limited to younger leaves only (Yu and De Luca, 2013). Similarly, spatial expression pattern has also been observed for other plant SM transporters including NtNUP1 (Hildreth et al., 2011), BUP1 (Dastmalchi et al., 2019), and CjABCB1/2 (Shitan et al., 2003, 2013) which were reported to majorly express specifically in root tips, latex, and rhizomes, respectively.

The role of chemical elicitors such as JA, MeJ, and salicylic acid (SA) has been well established in the enhanced production of several SMs including alkaloids, polyphenols, and terpenoids (De Geyter et al., 2012; Ho et al., 2020) which presumably also impact the activity of their respective transporters. For instance, BY-2 cells of *N. tabacum* displayed upregulation of nicotine-specific Nt-JAT1 transporter upon elicitation with MeJ. cDNA-amplified fragment length polymorphism-based transcript profiling indicates that the elicitation primarily induces the production of various nicotine biosynthesis enzymes. Further investigation by qRT-PCR in BY-2 cells and expression studies in tobacco seedlings also support the transcript-based data of enhanced expression of crucial biosynthesis genes, which is in coherence with strong induction of Nt-JAT1 transporter. Thus, the study precisely demonstrates the co-regulation of Nt-JAT1 with nicotine biosynthesis genes (Morita et al., 2009). On the other hand, expression of AtABCG40 transporter from *A. thaliana*, which is assumed to play role in transporting sclareol due to its functional similarity with other sclareol transporters, was observed to be positively enhanced upon elicitor treatments. *AtABCG40* displayed 260-fold induction at 12 h in response to SA while MeJ induces up to 40-fold after 6 h of treatment (Campbell et al., 2003). Similarly, fungal oligosaccharide when applied to the roots of *M. truncatula* could elevate the mRNA transcripts of MtABCG10, which plays an important role in defense response by modulating medicarpin biosynthesis in the roots of the plant (Banasiak et al., 2013). In addition, various plant growth regulators such as cytokinins, auxins, brassinosteroids, ethylene, and gibberellins are also reported to have a notable impact on the biosynthesis and accumulation of various nutrients or metabolites which in turn, might affect the functioning of corresponding transporters (Rawat et al., 2013; Jamwal et al., 2018). One such example is cytokinin-mediated substantial suppression of macronutrient (ammonium, nitrate, sulfate, and phosphate) transporters (Sakakibara et al., 2006). As reviewed by Jamwal et al. (2018) phytohormones are capable of significantly stimulating the SM production in the plant cell, for example, IBA, ethylene, kinetin, and GA_3_ enhances the biosynthesis of terpenoids, phenolics, glucosinolates, and steroidal lactones, respectively (Jamwal et al., 2018). As a result, it can be proposed that an increased metabolite pool should probably activate their transporters but only limited reports are available which state phytohormone mediated regulation of plant SM transporters. For example, auxins have been shown to enhance the expression of strigolactone biosynthesis genes of *P. hybrida*, which is possibly affecting the transporter function too. The transcript levels of *PhPDR1*, a strigolactone transporter were found to be increased in response to 1-naphthaleneacetic acid (NAA) treatment (Kretzschmar et al., 2012). Whereas the significance of ethylene in regulating the activity of AtABCG40 transporter was observed when pathogen-induced expression of *AtABCG40* transporter was significantly upregulated to 16-fold at 3 h after being treated with ethylene (Campbell et al., 2003). Moreover, the role of biotic stresses in transporter regulation is also evidently highlighted by significant upregulation of the glucosinolate transporters *GTR1* and *GTR2* of *B. rapa* in response to herbivory (Touw et al., 2020). Likewise, expression of *NpPDR1* is also found to be induced following infection with fungal (*Botrytis cinerea*) and bacterial (*Pseudomonas syringae*) pathogens via JA signaling pathway (Stukkens et al., 2005). The *Solanum tuberosum* leaves when infected with *Phytophthora infestans* showed upregulation of the *StPDR1/2/3/4* transporters, out of these, StPDR2 is proposed to be involved in the secretion of antifungal diterpene sclareol, which suggests the probable function of StPDRs in biotic stress responses. In addition, the cell suspension of *S. tuberosum* also showed upregulation of StPDR2 in response to the chemical treatment, which is certainly an indication of its role in abiotic stress tolerance (Ruocco et al., 2011). Similarly, abiotic factors such as metal stresses (Al, Mn, and Zn) are also reported to regulate the expression of *CcMATE34*, *CcMATE45*, and *CcMATE4* transporters of *Cajanus cajan* in a tissue-specific manner (Dong et al., 2019).

The presence of *cis*-regulatory elements (CRE) which are responsive toward various regulatory factors such as hormones, environmental conditions, biotic and abiotic stresses can control the expression of their respective genes at transcriptional levels. The common CREs have been identified in the promoter region of various potential ABC transporter genes of *Capsicum* and *Brassica* species (Yan et al., 2017; Lopez-Ortiz et al., 2019). CaABCC and CaABCG transporters of *Capsicum* contain light, heat, low temperature, and drought-responsive CREs (CCAATBOX1 and LTRECOREATCOR15) (Lopez-Ortiz et al., 2019). While, BraABC transporters from *Brassica* are found to have CREs that are responsive to various phytohormones including ABA, SA, GA (WRKY71OS and GT1CONSENSUS), pathogens (WRKY71OS), and wounding (WBOXNTERF3) (Yan et al., 2017). Additionally, the credit for enhanced transcription of *FaTT12-1* transporter of strawberry in response to red light irradiation has also been given to the occurrence of corresponding light-responsive CREs (Chen et al., 2018). Likewise, the promoter region of sclareol transporter NpPDR1 also possesses regulatory sequences, namely SB1 (sclareol box) (CACTAACACAAAGTAA) and SB3 (TTATGAACAGTAATTA) that are inducible in response to sclareolide, a close analog of sclareol. The mutation (nucleotide substitution) in the sequence of SB1 decreased the sclareolide mediated expression of *NpPDR1* by twofold while mutated SB3 almost completely diminished the transporter activity due to promoter inactivation. Further, it has also been observed that NpPDR1 which is otherwise positively induced becomes unresponsive toward MeJ upon mutating SB3, which suggests the role of SB3 as the transcriptional regulator in MeJ mediated induction of *NpPDR1* (Grec et al., 2003). Similarly, the role of transcription factors (TF) has also been well studied in context to the regulation of plant SMs. For example, the heterologous expression of *A. thaliana* MYB TF TT2 in the *M. truncatula* is reported to upregulate the *MtMATE1* which transports proanthocyanidin precursor epicatechin 3′-O-glucoside (E3′G) into the vacuole and further massive accumulation of E3′G in hairy roots confirms TF induced uptake by MtMATE1 transporter (Zhao and Dixon, 2009). Similarly, the LAP1-MYB type TF when constitutively expressed in *M. truncatula*, led to significant upregulation of *MtMATE2* transporter, which was primarily due to TF induced over production of anthocyanins (Zhao et al., 2011). Another transporter *SlMTP77* was also found to be upregulated in transgenic tomato lines overexpressing ANT1-MYB TF. In vegetative tissues, ANT1 lines caused strong purple pigmentation, which is due to increased biosynthesis of anthocyanidin that enhanced the activity of SlMTP77 (Mathews et al., 2003). Additionally, a genome-wide study in *S. lycopersicum* has examined the regulatory gene networks, which showed the importance of TFs and chromatin regulators as the master regulators for transcriptional control. The study has also identified a strong positive association of MYB28 TF and SQUAMOSA BINDING PROTEIN TF (SlySBP15) with potential MATE transporters of tomato (Santos et al., 2017). Furthermore, the transcriptional profile of VvMATE1 transporter in seed berries of *V. vinifera* was found to be positively regulated with the increase in expression of TFs namely VvMYBPA1 and VvMYBPA2 (Perez-Diaz et al., 2014). Several other TFs such as AaERF1/AaERF2 of *A. annua* (Yu et al., 2012), TSAR1/TSAR2 of *M. truncatula* (Mertens et al., 2016), and ORCA2/ORCA3 of *C. roseus* (Sun and Peebles, 2016) have been reported to play a role in regulating biosynthesis and accumulation of SM flux (Liu et al., 2017).

Like any other plant protein, the activity of transporters is also anticipated to be optimized and controlled by various post-translational modifications (PTM) such as phosphorylation, ubiquitination, acetylation, methylation, and glycosylation, etc. The role of phosphorylation and ubiquitination have been studied in various plant transporter proteins including potassium/nitrate/ammonium transporters and inorganic phosphate transporter (PHT1;4/PT2), respectively (Tang et al., 2020). In contrast, the activity of the NtPDR1 transporter, which exports sclareol and capsidiol out of the cell is presumed to be affected by its glycosylation at Asn 738 site (Pierman et al., 2017). However, more research is required to experimentally validate the significance of PTMs in context to the regulation of SM transporters. Yet, it seems reasonable to expect a similar mechanism by PTMs for SM transporters as already reported for various other plant proteins belonging to the same transporter families. For instance, the regulatory mechanisms of the ABCB1 transporter of *A. thaliana* are unveiled by its comparative study with a functionally characterized mammalian ABCC7 transporter which ultimately deciphered a conserved mechanism of regulation i.e., protein phosphorylation at serine residues by PINOID kinase. The addition of phosphate group occurs at S634 residue on the linker regulatory domain, which joins the NBD folds of the ABCB1 and such chemical modification is reported to modulate the function of the transporter. Besides, its negative regulation has also been observed when phosphorylation occurs at a different non-linker location (Aryal et al., 2015). Another important report from an auxin transporter ABCB19 of *A. thaliana*, also demonstrates phosphorylation mediated negative regulation by PHOTOTROPIN1 kinase (Christie et al., 2011). Additionally, the chemical modification of SMs might also enhance their potential as a substrate which tends to have a profound effect on their uptake by respective transporters (Le Roy et al., 2016). Various plant SM transporters exclusively utilize modified substrates or prefer them with higher affinity. Specifically, the flavonoids undertake various chemical modifications including glycosylation, acylation, methylation, or hydroxylation, to get recognized by their transporters (Zhao and Dixon, 2010). As reported for the AtABCC2 transporter, the cyanidin 3-O-glucoside anthocyanins are the exclusive substrates over their aglycon variants (Behrens et al., 2019). Furthermore, AM1 and AM3 transporters from *V. vinifera* selectively utilize acylated anthocyanins as substrate (Gomez et al., 2009), whereas MtMATE2 prefers malonylated flavonoids over the non-malonylated form (Zhao et al., 2011).

A large number of plant SMs such as terpenoids, phenolics, alkaloids, tannins, and many others possess antioxidant potential (Wink, 2015; Rehman and Khan, 2017). While several other SMs also function as redox modulatory compounds such as organic isothiocyanates from the Brassicaceae family and polysulfanes from *Allium* genus (Jacob et al., 2011). As observed for primary metabolites such as sugars, the transport activity is well known to be regulated by the redox status of the cell (Griffiths et al., 2016). Therefore, it can be postulated that the altered redox status of a cell might also possibly have an impact on SM accumulation and transport. However, unlike primary metabolites, this hypothesis lacks scientific validation for redox-regulated transport of SMs.

## Modulation of Secondary Metabolite Transport and Its Implication in Transporter Engineering

**FIGURE 4:**
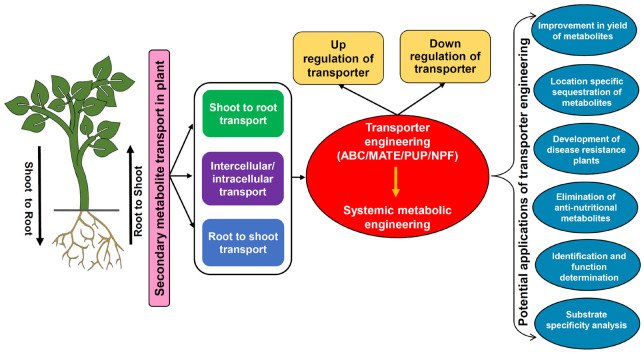
The transporter engineering model depicting the scheme for modulation in the expression of transporters and their applications. ABC, ATP binding cassette; MATE, multidrug and toxic compound extrusion; NPF, nitrate and peptide transporter family; PUP, purine uptake permease.

Secondary metabolites are extremely diverse from a structural as well as functional standpoint hence modulation in their transport should have significant effects on a complex array of plant processes. Engineering SM transporters by modulating their expression/activity will pave the way for enhancing metabolite production, tissue/organ-specific accumulation, biotic and abiotic stress tolerance, reducing anti-nutritional compounds, identification of transporters with novel functions, and their characterization (Figure 4; Nour-Eldin and Halkier, 2013; Jorgensen et al., 2015; Lv et al., 2016). The fine-tuning of transporter expression needs to be executed in accordance with the regulatory factors so that overall plant growth and other basic functions linked to SMs are not hampered. Further, the development of an efficient system for transporter engineering also remains challenging due to limitations involved in the functional characterization and substrate specificity determination for most transporters (Larsen et al., 2017a). Although due to ligand specificity, few of the transporters might mimic enzymes if they recognize only a particular SM, yet in most cases, transporters can bind with multiple substrates. Therefore, modulating the expression of a specific transporter will also possibly disturb the unknown plant processes hence the selection of an appropriate transporter whose functional specificities have very well been determined is the key requisite. So far, limited reports are available for the implementation of transporter engineering by altering the accumulation and transport of important SMs. However, it is indeed a promising approach for enhanced production of desirable SMs that is plausibly attainable if the organ-specific sequestration inside the cells or export and accumulation into the culture medium can be regulated systematically to amend the control of metabolite flux (Kell et al., 2015). The below discussed examples highlight prospects of transporter engineering in context to various plant SMs.

A recent study in *C. roseus* demonstrated that a fivefold higher accumulation of catharanthine was achieved in hairy roots by overexpression of the *CrTPT2* transporter, which was otherwise unattainable by conventional strategies (Wang et al., 2019). This directed the translocation of catharanthine metabolite in a regulated manner from its synthesis site hairy roots to the vacuole or intercellular space for storage. Therefore, the possibility of negative feedback inhibition was also prevented that might have happened due to excess accumulation at the synthesis site and eventually promoted enhanced yield of catharanthine in the sink tissue. In contrast, another transporter of *C. roseus CrNPF2.9* upon downregulation showed significantly reduced transport of strictosidine from leaf vacuole to cytosol, which led to organ-specific accumulation of strictosidine only in the leaf vacuole (Payne et al., 2017). Further, modulation in transporter expression also has an impact on the response of plants toward various biotic stresses because it ultimately affects the accumulation of various SMs that are important components of the plant immune system (Isah, 2019). For example, the MtABCG10 transporter of *M. truncatula* transports precursors for the synthesis of medicarpin in the hairy roots of the plant (Biala et al., 2017). Medicarpin is the major phytoalexin of *Medicago*, which is known to participate in defense responses, particularly against fungal pathogens. The RNAi-mediated silencing of the *MtABCG10* gene resulted in a compromised defense response against root infecting fungi *Fusarium oxysporum* (Banasiak et al., 2013), thus it can be postulated that knockdown of transporter expression caused reduced translocation of isoflavone medicarpin precursors hence disease resistance was found to be hampered. A similar response was also observed in *N. plumbaginifolia* where silencing of *NpPDR1* transporter made the plants more sensitive toward various fungal and oomycete pathogens (Bultreys et al., 2009). On the other hand, Nb-ABCG1/2 transporters of *N. benthamiana* are essential in providing resistance against a pathogen *P. infestans* due to their involvement in the transport of defense compound capsidiol to the site of pathogen attack (Shibata et al., 2016). Thus, it can be expected that the engineering of SM transporters having a vital role in defense mechanisms holds great potential toward the production of disease-resistant crops. Similarly, many transporters are also known to play important role in abiotic stress tolerance such as drought resistance. The members of ABCG subfamily have been known to transport wax toward the surface of the plant, which eventually reduces the water loss due to its lipophilic properties (Shitan et al., 2013). Another commonly used drought tolerance mechanism by plants is the utilization of ABA phytohormone to attain stomatal closure under water deficit conditions. Thus, the contribution of transporters in regulating ABA influx/efflux is worth exploring and one such example is AtABCG25 exporter of *A. thaliana*, which participates in the intercellular distribution of ABA, whereas for the import of extracellular ABA, plasma membrane-localized AtABCG40 transporter is needed which is reported to act against drought stress (Kang et al., 2010; Dahuja et al., 2021). Studies also suggest that the biosynthesis and transport of SMs get affected under drought stress. Specifically, to fight against drought-induced oxidative stress, defense-related SMs such as phenolics and terpenes are needed to be increased in the cell (Kumar et al., 2021). Therefore, regulating the transport and accumulation of SMs linked with drought stress tolerance also seems to be achievable by transporter engineering approaches. Although much information is not available but some of the recent studies in cassava (Yan Y. et al., 2021) and grapevine (Tu et al., 2020) have proposed that enhanced lignin accumulation is also positively linked with drought resistance, which opens up the possibility of modulating the lignin transporters to increase its deposition in the cell wall. Altogether, drought tolerance using transporter modulation should have a positive impact on reducing crop losses in the agricultural sector.

The significance of transporter modulation in removal and/or reduction of the anti-nutritional metabolites from valuable plant parts has been reported in *A. thaliana* (Nour-Eldin et al., 2012) and *Brassica* species (Nour-Eldin et al., 2017). This strategy will surely increase the nutritional value of the crops by preventing/reducing the accumulation of toxic SMs in the edible plant parts. The expression of AtGTR1 and AtGTR2 transporters of *A. thaliana* was significantly reduced by generating their double mutants via DNA mutagenesis (Nour-Eldin et al., 2012), which led to the negligible accumulation of glucosinolates in seeds while their source tissues such as leaves and silique wall have shown more than 10-fold higher accumulation. Further, Nour-Eldin et al. (2017) have attempted to translate a similar strategy from *A. thaliana* to even more challenging and complex polyploid *Brassica* species, which are globally utilized as oilseed crops but suffer from low nutritional content due to the accumulation of glucosinolates in their seeds. Downregulation of GTR transporters from *B. rapa* (*BrGTR2*) and *Brassica juncea* (*BjGTR2*) was achieved by inserting early stop codons in their genes via mutagenesis and this facilitated a more than 60% reduction in the accumulation of seed glucosinolates (Nour-Eldin et al., 2017). The reduction of glucosinolates from edible seeds has also been attempted previously using transgenic approaches (Augustine et al., 2013) and interspecific crossing (Burton et al., 2003) only with limited success. Therefore, the remarkable outcomes of modulating *Brassica* GTR transporters have certainly validated the potential of transporter engineering in agriculturally important plants. These genetic manipulations are preferred for reducing glucosinolate contents over classical post-harvest processing of crops. From an agrobiotechnology perspective, post-harvest techniques are quite demanding in terms of cost and time, and on the other side, these might also affect protein digestibility and solubility in the meal. It has also been proposed that *B. juncea* with low levels of seed glucosinolates might also be suitable for arid regions (Burton et al., 2003). The identification of GTRs opens the avenue for many other crops where the distribution of anti-nutritional compounds is under the control of transport proteins.

Besides engineering the expression of the gene, modulation in the activity of the transporter protein by other means can also aid in the functional characterization and substrate specificity analysis. If the preliminary information of unknown transporters can be obtained by bioinformatic tools then their further identification can be performed either by providing exogenous substrates/analogs to upregulate or the chemical inhibitors to downregulate the activity of the transporters that will certainly facilitate functional validation. For instance, the expression studies of *C. japonica CjABCB1* in the *Xenopus* oocyte system revealed that treatment with specific chemical agents has significantly inhibited the transporter activity (Shitan et al., 2003). At the same time, uptake of the proposed substrate berberine was also drastically diminished, which indicated that CjABCB1 is a berberine-specific transporter that enables its translocation into the rhizome. Similarly, the substrate specificity determination for NtNUP1 transporter of *N. tabacum* was conducted by expressing cDNA of *NUP1* gene in *S. pombe* and the transformed cells were analyzed for the uptake of radiolabeled substrates. Further, the uptake assays demonstrate that ^14^C-nicotine accumulation was significantly high in the case of *NUP1* expressing cells relative to control cells (expressing empty vector) (Hildreth et al., 2011). As previously discussed in sections “ATP Binding Cassette Transporters,” “Multidrug and Toxic Compound Extrusion Transporters,” “Purine Uptake Permease Transporters,” “Nitrate and Peptide Transporter Family Transporters,” several proteins have also been discovered for their transport abilities by studying the effects of their altered expression levels. One such example is *AaPDR3* transporter whose overexpression has shown an increase in the β-caryophyllene content while downregulation led to its reduced accumulation, which has identified AaPDR3 as a transporter for β-caryophyllene (Fu et al., 2017).

## Conclusion and Future Perspectives

Metabolic engineering of plants has been given a lot of importance in recent years due to its immense potentials. Various strategies have been deciphered to increase the yield of SMs and their accumulation. In the majority of earlier research efforts, the increase in metabolite yield was achieved through the modulation of the biosynthetic pathway genes. However, advancement of knowledge in the area opens possibilities of regulation of potential transporter genes for precise metabolic engineering. The transporters of plant SMs are the key players that have an impact not just at the site of metabolite synthesis and accumulation but systemically affect the plant metabolic system in one way or the other. In the recent past, several SM transporters have been identified in plants but their functional validation and substrate specificity determination is a huge challenge and equally critical for the metabolic engineering of plants. The present review emphasizes the knowledge gain in the area concerning SM transporters and their modulation. The transporter engineering, particularly for plant stress (biotic and abiotic) tolerance, SM accumulation, and removal of anti-nutritional compounds will be of great future potential. Further, the leverage of this technology can be extended to many medicinal, agricultural, aromatic, and other economically important plants for their optimum utilization and value addition. However, for efficient transporter engineering, more emphasis is required to identify the possible involvement of multiple transporters and/or their cross-talks for the long-distance mobilization of SMs. In addition, more and more transporters associated with secretion and storage should be annotated to get better insight into their functionality and possible applications. Similarly, the workings of the two-way transport system and the source-sink relationship linked to the distribution of SM pool need to be investigated in detail. Overall, it is realized that transport engineering is a dynamic and fairly complex process and emerges as a critical tool for systemic metabolic engineering in plants. However, it is also equally important to address the associated challenges using modern experimental tools and approaches to make it more effective and harness its fullest potential.

## Author Contributions

PN wrote the original draft. PP conceptualized the idea, supervised, and critically reviewed the manuscript. Both authors significantly contributed to finalizing the manuscript and approved it for submission.

## Conflict of Interest

The authors declare that the research was conducted in the absence of any commercial or financial relationships that could be construed as a potential conflict of interest.

## Publisher’s Note

All claims expressed in this article are solely those of the authors and do not necessarily represent those of their affiliated organizations, or those of the publisher, the editors and the reviewers. Any product that may be evaluated in this article, or claim that may be made by its manufacturer, is not guaranteed or endorsed by the publisher.
