# Supplementary material for: Plant Secondary Metabolite Transporters: Diversity, Functionality, and Their Modulation
Source: Front Plant Sci. 2021 Oct 27;12:758202. doi: 10.3389/fpls.2021.758202 (PMC8580416; doi:10.3389/fpls.2021.758202)
Supplement: Supplementary file 1 [file Table_1.doc]

**Table S1**: Salient features of selected plant secondary metabolite transporters

| Sr.  No. | Plant species | Transporter | Transp-orter family/sub-family | Probable localization (membrane/ tissue/organ) | Approaches used for localization determination | Proposed function of transporter | Approaches used for function identification/  characterization | Class of secondary metabolites | Reference |
| --- | --- | --- | --- | --- | --- | --- | --- | --- | --- |
| **ATP binding cassette transporters (ABC)** | | | | | | | | | |
|  | *Arabidopsis thaliana*  (Thale cress) | AtABCC2 | ABCC | *Tonoplast/ vegetative tissues | Membrane (vacuolar) enriched vesicles | Vacuolar sequestration of anthocyanins (cyanidin 3-O-glucoside) and other flavonoids | Molecular modeling, ligand docking, expression, and *in vitro* uptake assays in yeast mutants | Flavonoid | Behrens et al. (2019) |
|  | *Arabidopsis thaliana*  (Thale cress) | AtABCG29 | ABCG | Plasma membrane/root endodermis and stem | YFP fusion, GFP fusion | Transport of monolignol (p-coumaryl alcohol) from the cytosol to the cell wall | Expression in yeast mutants, transport assays with yeast microsomes, study of knockout mutants | Phenolic compounds | Alejandro et al. (2012) |
|  | *Arabidopsis thaliana*  (Thale cress) | AtABCG34 | ABCG | Plasma membrane/ epidermal cells of roots and leaves | GFP colocalization | Secretion of camalexin from the leaf surface | Analysis in *A. thaliana* overexpression lines and *N. tabacum* BY-2 cells | Indole alkaloid | Khare et al. (2017) |
|  | *Arabidopsis thaliana*  (Thale cress) | AtABCG37 | ABCG | Plasma membrane/root | Immunostaining, GFP colocalization, qRT-PCR using root samples | *Secretion of scopoletin (coumarin) and its derivatives from roots | Detection of scopoletin secretion in the medium by HPLC-MS/MS, expression analysis by qRT-PCR, ABCG37 promoter analysis | Phenolic compounds | Fourcroy et al. (2014); Ruzicka et al. (2010) |
|  | *Arabidopsis thaliana*  (Thale cress) | AtABCG40  (AtPDR12) | ABCG | *Plasma membrane | Prediction by PSORT (amino acid sequence analysis) | *Export of sclareol from cells | Expression analysis by qRT-PCR, AtPDR12 T-DNA insertion line analysis, theoretical analogy with NpABC1 and SpTUR2 | Diterpene | Campbell et al. (2003) |
|  | *Artemisia annua*  (Sweet wormwood) | AaPDR3  (TPT2) | ABCG/PDR | Plasma membrane/T-shaped trichomes of old leaves and roots, stem, flower buds | Prediction by PREDOTAR and WoLF PSORT, GFP colocalization, GUS assay | Cellular uptake of β-caryophyllene | Expression in yeast cells and uptake assays, qRT-PCR analysis in RNAi, and overexpression transgenic lines | Sesquiterpene | Fu et al. (2017) |
|  | #*Capsicum* species  (Pepper) | unidentified | ABCC and ABCG | Tonoplast/ placental epidermal cells | Theoretical analogy with other ABC transporters | *Vacuolar uptake of capsaicin and dihydrocapsaicin | Expression analysis using RNA-Seq data and by qRT-PCR, theoretical analogy | Alkaloid | Lopez-Ortiz et al. (2019) |
|  | *Catharanthus roseus*  (Periwinkle) | CrTPT2 | ABCG/PDR | Plasma membrane/ epidermal cells of young leaves | GFP fusion, transient expression in yeast and onion epidermal cells | Efflux of catharanthine to the leaf surface | Virus-induced gene silencing, expression in yeast cells, *in vitro* substrate specificity assays | Alkaloid | Yu and De Luca (2013) |
|  | *Coptis japonica*  (Japanese goldthread) | CjABCB1 (CjMDR1) | ABCB | Plasma membrane/ rhizome, flower petiole, and peduncle | Northern hybridization, membrane fractionation, immunoblotting, *in situ* hybridization | Influx of berberine to rhizome from root | Expression in *Xenopus* oocyte and substrate uptake activity assays, drug sensitivity assay in yeast cell | Alkaloid | Shitan et al. (2003); Yazaki et al. (2001) |
|  | *Coptis japonica*  (Japanese goldthread) | CjABCB2 | ABCB | Plasma membrane/ exclusively in the rhizome | Northern hybridization, membrane fractionation, *in situ* hybridization | Influx of berberine to rhizome from root | Expression in cell culture of *C. japonica*, plant organs, and yeast cells | Alkaloid | Shitan et al. (2013) |
|  | *Coptis japonica*  (Japanese goldthread) | CjABCB3 | ABCB | unidentified | - | *Influx of berberine only in cultured plant cell | Expression in yeast cells and theoretical analogy with other similar transporters | Alkaloid | Shitan et al. (2013) |
|  | #*Coptis deltoidea*  (Goldthread) | unidentified | ABCB | *Roots (higher expression) and rhizome | Indirect correlation by UHPLC-ESI-MS/MS analysis | *Transport of benzylisoquinoline alkaloids | Transcriptome analysis, qRT-PCR, theoretical analogy | Alkaloid | Zhong et al. (2020) |
|  | *Crocus sativus*  (Saffron) | CsABCC4a | ABCC | Tonoplast/stigma | Infiltration of *N. benthamiana* leaves, GFP fusion, co-expression analysis, TMHMM | Transport of crocins from cytosol to vacuole | Co-expression analysis, expression in yeast cells, transportomic assays in yeast microsomal cells | Apocarotenoid glycoside | Demurtas et al. (2019) |
|  | #*Gossypium hirsutum* (Brown cotton) | GhABCC24, GhABCC27, GhABCC28, GhABCC29, and GhABCC33 | ABCC | *Tonoplast/ cotton fiber | Predicted by Cell-PLoc 2.0 software | *Probable role in transport of proanthocyanidin | Whole-genome analysis, phylogenetic and theoretical analogy with other similar transporters, qRT-PCR | Flavonoid | Sun et al. (2021) |
|  | *Medicago truncatula*  (Barrel medic) | MtABCG10 | ABCG | Plasma membrane/root (highest), leaves, flower, and fruits | *In situ* immuno- localization, membrane fractionation, GUS reporter analysis, GFP fusion | Transport/  distribution of 4-coumarate and liquiritigenin (medicarpin precursors) in roots | Analysis of RNAi silenced transgenic hairy roots, qRT-PCR, expression in *N. tabacum* BY-2 cells, metabolite estimation (HPLC/MS) | Isoflavonoid | Banasiak et al. (2013);  Biala et al. (2017) |
|  | *Nicotiana benthamiana*  (Wild tobacco) | Nb-ABCG1,  and  Nb-ABCG2 | ABCG | Plasma membrane/leaves | Transient expression of GFP fusion in *N. benthamiana* leaves | Secretion of capsidiol outside of the cell | Analysis of VIGS based transgenic plants, qRT-PCR | Sesquiterpene | Shibata et al. (2016) |
|  | *Nicotiana* *plumbaginifolia*  (Tex-Mex tobacco) | NpPDR1  (NpABC1) | ABCG | Plasma membrane/  whole root, leaf glandular trichomes, flower petals | Membrane fractionation, *in vitro* and in situ immunodetection, GUS histochemical assay | Transport of sclareol and probably other related diterpenes to the leaf surface | Expression analysis in leaves and cell suspension culture, RNAi transgenic line analysis, sclareol toxicity assays | Diterpene | Jasiński et al. (2001); Stukkens et al. (2005) |
|  | *Nicotiana tabacum*  (Tobacco) | NtPDR1 | ABCG | Plasma membrane/root epidermis, leaf, flower, glandular trichomes | Membrane fractionation, in situ immuno- localization, GUS histochemical assay | Secretion of diterpenes (sclareol) and sesquiterpene (capsidiol) outside of the cell | Overexpression in BY-2 suspension cells, substrate transport assay | Terpene | Crouzet et al. (2013); Pierman et al. (2017); Sasabe et al. (2002) |
|  | *Nicotiana tabacum*  (Tobacco) | NtPDR6 | ABCG | *Roots | GUS histochemical assay | *Probably involved in transport of strigolactone | Phylogenetic analysis, theoretical analogy, and sequence similarity with PhPDR1 | Carotenoid derived phytohormone | Xie et al. (2015) |
|  | *Panax ginseng*  (Ginseng) | PgPDR3 | ABCG | *Roots and adventitious roots, leaves | RT and qRT-PCR | *Accumulation and transport/export of ginsenosides | RT and qRT-PCR | Triterpene saponins | Cao et al. (2015); Zhang et al. (2013) |
|  | *Petunia hybrida*  (Garden petunia) | PhPDR1 | ABCG | Plasma membrane/stem and nodal tissue | Transient expression of GFP fusion in *A. thaliana*, qRT-PCR, GUS assay | Cellular export of strigolactone, mostly from roots | Mutant analysis, study of RNAi transgenic lines, overexpression in *A. thaliana*, transport assay | Carotenoid derived phytohormone | Kretzschmar et al. (2012) |
|  | *Petunia hybrida*  (Garden petunia) | PhABCG1 | ABCG | Plasma membrane/petals of open flowers | Membrane specific marker and microsomal fractions | Export of volatile organic compounds from flowers | Study of RNAi transgenic lines, expression and transport assays in *N. tabacum* BY-2 cells | Volatile organic compounds | Adebesin et al. (2017) |
|  | *Salvia miltiorrhiza*  (Danshen) | SmABCC1 | ABCC | *Flowers, leaves, stems, roots (highest expression) pericytes, phloem, xylem | Transcriptome analysis, qRT-PCR | *Probable role in the transport of tanshinone and salvianolic acid | Co-expression analysis with biosynthesis pathway genes, phylogenetic analysis | Diterpenoids (tanshinone) and phenolic acids (salvianolic acid) | Yan et al. (2021) |
|  | *Salvia miltiorrhiza*  (Danshen) | SmABCG46, SmABCG40, and SmABCG4 | ABCG | *Mostly in all organs/tissue with highest expression in root (plasma membrane/ SmABCG46) and leaf (SmABCG40 and SmABCG4) | Transcriptome analysis, qRT-PCR, GFP fusion studies in transiently transformed tobacco leaves (SmABCG46) | *Probable role in the transport of tanshinone and salvianolic acid | Co-expression analysis with biosynthesis pathway genes, phylogenetic analysis | Diterpenoids and phenolic acids | Yan et al. (2021) |
|  | *Spirodela polyrrhiza*  (Duckweed) | SpTUR2 | ABCG | Plasma membrane | Membrane fractionation, immuno- localization | *Transport of sclareol out of plant tissue | Expression in *A. thaliana*, GC-MS analysis of *Spirodela* extracts, northern blot analysis | Diterpene | van den Brule et al. (2002) |
|  | *Solanum tuberosum*  (Potato) | StPDR1, StPDR2, StPDR3, and  StPDR4 | ABCG | *Roots, leaves, tubers and stem | qRT-PCR | *StPDR2 could be involved in sclareol transport | Exogenous treatment of substrates followed by qRT-PCR, theoretical analogy with SpTUR2, NpABC1, and AtPDR12 | Diterpene | Ruocco et al. (2011) |
|  | *Tripterygium wilfordii* Hook  (Thunder god vine) | TwPDR1 | ABCG | Plasma membrane/ adventitious roots | GFP fusion study in transiently transformed onion epidermal cells, qRT-PCR | Efflux of triptolide | Study of RNAi and overexpression plants and tobacco BY-2 protoplasts | Diterpene triepoxide | Miao et al. (2021) |
|  | *Vitis vinifera* (Grapevine) | VvABCC1 | ABCC | Tonoplast/  Exocarp and mesocarp, old leaves and berries | Transient expression of GFP fusion in *N. benthamiana*, qRT-PCR | Transport of malvidin 3-O-glucoside into exocarp vacuole | Expression in yeast vesicle cells, transport uptake assay, phylogenetic analysis | Flavonoid | Francisco et al. ( 2013) |
|  | *Zea mays*  (Maize) | ZmMRP3 | ABCC | Tonoplast/shoot, developing adult leaf, ear, and tassel | GFP fusion, northern blot | *Transport of anthocyanin into the vacuole | Study of mutant (antisense) transgenic plants, HPLC analysis | Flavonoid | Goodman et al. (2004) |
| **Multidrug and toxic compound extrusion transporters (MATE)** | | | | | | | | | |
|  | *Arabidopsis thaliana*  (Thale cress) | AtFFT (DTX35) | MATE | *Tonoplast/seed coat, meristematic root cells, sub-epidermal cells of hypocotyls | GFP fusion | *Accumulation of anthocyanin in immature seed | Amino acid sequence similarity with other similar transporters, RT-PCR, mutant complementation | Flavonoid | Kitamura et al. (2016) |
|  | *Arabidopsis thaliana*  (Thale cress) | AtTT12 | MATE | *Tonoplast and vesicular membranes/ endothelium layers of immature seeds and ovules | qRT-PCR | *Transport of proanthocyanidin precursors (catechin and leucocyanidin) into vacuoles | Amino acid sequence similarity with other similar transporters, staining and microscopic analysis in mutants | Flavonoid | Debeaujon et al. (2001) |
|  | #*Cajanus cajan* (Pigeonpea) | CcMATE34 and CcMATE45 | MATE | *Roots and stem (CcMATE34); roots, stem, leaves, and flowers (CcMATE45) | qRT-PCR | Probable role in the transport of alkaloids | Whole-genome analysis, phylogenetic and theoretical analogy with other similar transporters | Alkaloid | Dong et al. (2019) |
|  | #*Cajanus cajan* (Pigeonpea) | CcMATE4 | MATE | *Roots | qRT-PCR | *Probable role in transport of flavonoids | Whole-genome analysis, phylogenetic and theoretical analogy with other similar transporters | Flavonoid | Dong et al. (2019) |
|  | #*Coptis deltoidea*  (Goldthread) | unidentified | MATE | *Roots and rhizome (higher expression) | Indirect correlation by UHPLC-ESI-MS/MS analysis | *Transport and vacuolar accumulation of benzylisoquinoline alkaloids | Transcriptome analysis, qRT-PCR, and theoretical analogy | Alkaloid | Zhong et al. (2020) |
|  | *Coptis japonica*  (Japanese goldthread) | CjMATE1 | MATE | Tonoplast/  rhizome, petiole, root, and leaf | GFP fusion and qRT-PCR | Transport of berberine into vacuole of cultured cells | Cellular transport assays using yeast strain | Alkaloid | Takanashi et al. (2017) |
|  | #*Fagopyrum esculentum*  (Buckwheat) | FeTT12 | MATE | *Leaves | qRT-PCR | *Translocation of proanthocyanidin | Phylogenetic and theoretical analogy with other similar transporters | Flavonoid | Chang et al. (2018) |
|  | *Fragaria* x *ananassa*  (Strawberry) | FaTT12-1 | MATE | *Fruits, leaves, roots, crown, stolon | qRT-PCR | Accumulation of proanthocyanidin in fruits | Phylogenetic analysis and virus-induced gene silencing | Flavonoid | Chen et al. (2018) |
|  | *Gossypium hirsutum* (Upland cotton) | GhTT12 | MATE | *Tonoplast/cotton fiber | GFP fusion studies in transiently transformed tobacco, qRT-PCR | *Transport of proanthocyanidin (PA) from the cytoplasm to vacuole | Phylogenetic analysis, PA content determination, qRT-PCR, expression in stably transformed *A. thaliana* lines | Flavonoid | Gao et al. (2016); Xu et al. (2019) |
|  | #*Gossypium hirsutum* (Upland cotton) | GhMATE12, GhMATE16, and GhMATE38 | MATE | *Tonoplast/  cotton fiber | Theoretical analogy with GhTT12 transporter | *Transport of proanthocyanidin from the cytoplasm to vacuole | Whole-genome and phylogenetic analysis, qRT-PCR, and theoretical analogy | Flavonoid | Xu et al. (2019) |
|  | *Malus* x domestica (Apple) | MdMATE1, and MdMATE2 | MATE | *Tonoplast/fruit and leaves | qRT-PCR, theoretical analogy with AtTT12 transporter | *Transport of proanthocyanidin from the cytosol to vacuole | Phylogenetic analysis, PA content determination, mutant complementation | Flavonoid | Frank et al. (2011) |
|  | *Medicago truncatula*  (Barrel medic) | MtMATE1 | MATE | *Tonoplast/ flowers, young pods, seed coat | GFP fusion studies in transiently transformed tobacco, qRT-PCR | Transport of proanthocyanidin precursors (epicatechin 3'-O-glucoside) into vacuoles | PA accumulation studies in *Medicago* hairy roots, mutant complementation, loss of function studies | Flavonoid | Zhao and Dixon (2009) |
|  | *Medicago truncatula* (Barrel medic) | MtMATE2 | MATE | Tonoplast/leaves, flowers, roots, vegetative buds, and seeds | Microarray data, qRT-PCR, in situ hybridization, GFP fusion study in plant and yeast cells | Transport of anthocyanin (malonylated  flavonoid glucosides) into vacuole | Expression in yeast cell microsomes, substrate uptake assay, loss of function studies | Flavonoid | Zhao et al. (2011) |
|  | *Nicotiana tabacum*  (Tobacco) | NtMATE1, and NtMATE2 | MATE | Tonoplast/roots and flowers | GFP fusion, GUS assay, membrane fractionation, immunogold electron microscopy | Transport of nicotine and related alkaloids to the vacuolar lumen of roots | Expression in yeast cells, *in vitro* transport assay, RNAi, and overexpression transgenic plant analysis | Alkaloid | Shoji et al. (2009) |
|  | *Nicotiana tabacum* (Tobacco) | Nt-JAT1 | MATE | Tonoplast/leaves, roots, flowers, and stems (*Probably dual localization at leaf tonoplast and root plasma membrane ?) | Membrane fractionation, immunodetection, RNA gel blot analysis | Transport of nicotine and related alkaloids to the vacuolar lumen of leaves | Transcript profiling, expression in yeast cells with substrate transport assay, proteoliposome based *in vitro* transport assays | Alkaloid | Morita et al. (2009); Shitan et al. (2009) |
|  | *Nicotiana tabacum*  (Tobacco) | Nt-JAT2 | MATE | Tonoplast/leaves | Study of GFP fusion in tobacco BY-2 cells, RNA gel blot analysis | Transport of nicotine and related alkaloids to the vacuolar lumen of leaves | Phylogenetic analysis, expression in yeast cells with substrate transport assay | Alkaloid | Shitan et al., (2015); Shitan et al. (2014) |
|  | #*Oryza sativa*  (Black rice) | OsMATE34 | MATE | *Vacuole/  caryopsis cells | Predicted by TMHMM online server | *Probable role in transport of anthocyanin from cytoplast to vacuole | RNA-Seq analysis, phylogenetic studies (theoretical analogy with other plants), qRT-PCR | Flavonoid | Mackon et al. (2021) |
|  | #*Raphanus sativus* L. (Radish) | RsMATE2-3 RsMATE5, RsMATE7-9 | MATE | *Plasma membrane/  leaves, roots, and stem | Prediction by PSORT, qRT-PCR, theoretical analogy | *Transportation of anthocyanin | Transcript profiling, phylogenetic analysis, qRT-PCR, theoretical analogy | Flavonoid | M’mbone et al. (2018) |
|  | #*Vaccinium corymbosum*  (Blueberry) | VcMATE2, VcMATE3,  VcMATE5,  VcMATE7, VcMATE8,  and  VcMATE9 | MATE | *Plasma membrane/fruit exocarp and roots | Prediction by PSORT, qRT-PCR, theoretical analogy | *Transport of anthocyanin, pro- anthocyanidine, and flavonols | Transcriptome analysis, phylogenetic analysis, and theoretical analogy | Flavonoid | Chen et al. (2015) |
|  | *Vitis vinifera* (Grapevine) | AM1, and AM3 | MATE | Tonoplast and nucleus attached membranes of hairy root epidermal cells /berry skin, leaves | GFP fusion study in stably transformed hairy roots, qRT-PCR | Transport of acylated anthocyanin from the cytosol to vacuole | Phylogenetic analysis, expression in yeast microsomal vesicles, inhibitor studies, transport activity assays | Flavonoid | Gomez et al. (2009) |
|  | *Vitis vinifera* (Grapevine) | VvMATE1 | MATE | Tonoplast/seed berry | GFP fusion study in *A. thaliana* protoplast, prediction by Wolf PSORT-II | *Accumulation of proanthocyanidine in the vacuole | Phylogenetic analysis, qRT-PCR, and theoretical analogy | Flavonoid | Perez-Diaz et al. (2014) |
|  | *Vitis vinifera* (Grapevine) | VvMATE2 | MATE | Plasma membrane/  golgi complex; seed berry and leaves | GFP fusion study in *A. thaliana* protoplast, qRT-PCR, prediction by WoLF PSORT-II | *Translocation of proanthocyanidine in apoplast/cell wall | Phylogenetic analysis, qRT-PCR, and theoretical analogy | Flavonoid | Perez-Diaz et al. (2014) |
|  | *Solanum lycopersicum*  (Tomato) | SlMTP77 | MATE | *Tonoplast/  leaves | Theoretical analogy with AtTT12, study of ANT1 tomato mutant (indirect correlation) | *Transport of anthocyanin into leaf vacuole | RNA gel blot and SSH analysis, study of ANT1 tomato mutant (indirect correlation), theoretical analogy | Flavonoid | Mathews et al. (2003) |
| **Purine uptake permease transporters (PUP)** | | | | | | | | | |
|  | *Nicotiana tabacum*  (Tobacco) | NtNUP1 | PUP like transpo-rters | Plasma membrane/roots (specifically root tips) and leaves | Study of GFP fusion in *N. tabacum* BY-2 protoplasts, qRT-PCR | Uptake of nicotine from the apoplast to the cytoplasm of root cells | Study of RNAi transgenic plants and hairy root cultures, expression in yeast cells, transport activity assays | Alkaloid | Hildreth et al. (2011); Kato et al. (2015) |
|  | *Papaver somniferum* (Opium poppy) | BUP1 | PUP like transpo-rters | Plasma membrane/ laticifers cells | GFP fusion study in transiently transformed onion epidermal cells, qRT-PCR | Import of opiate alkaloids to laticifers cells from sieve elements | Transcriptome and phylogenetic analysis, expression in yeast cells, substrate uptake assays, virus-induced gene silencing | Benzylisoquin-oline alkaloids | Dastmalchi et al. (2019) |
| **Nitrate and peptide transporter (NRT/PTR or NPF)** | | | | | | | | | |
|  | *Arabidopsis thaliana*  (Thale cress) | AtGTR1 (NPF2.10), and AtGTR2 (NPF2.11) | NPF | Plasma membrane/leaves and roots | YFP fusion and GUS assay study in stable transgenic lines, micro-grafting | Long-distance transport of aliphatic glucosinolates from leaves to seeds; rosette to roots (bidirectional transport via xylem and phloem) | Phylogenetic analysis, expression in *Xenopus* oocytes, study of mutants, HPLC analysis, reciprocal micro-grafting | Glucosinolates | Andersen et al. (2013); Nour-Eldin et al. (2012) |
|  | *Arabidopsis thaliana*  (Thale cress) | AtGTR3  (NPF2.9) | NPF | Plasma membrane/root phloem cells | YFP fusion, micro-grafting | Involved in transport of indole glucosinolates between root and rosette | Phylogenetic analysis, expression in *Xenopus* oocytes, reciprocal micro-grafting, LC-MS analysis, transport activity assay | Glucosinolates | Jørgensen et al. (2017) |
|  | *Brassica juncea*  (Oilseed) | BjuGTR1 and  BjuGTR2 | NPF | *All the vegetative and reproductive tissues | qRT-PCR | Transport of glucosinolates possibly from leaves to seeds | Expression studies in GTR-deficient mutant lines and RNAi plants, plant based transport assay | Glucosinolates | Nambiar et al. (2021) |
|  | *#Brassica napus*  (Rapeseed) | BnaNPF2.12, BnaNPF2.13, BnaNPF2.14,  BnaNPF2.19, BnaNPF2.20, BnaNPF2.26, and BnaNPF2.28 | NPF | *Vacuole or  Plasmalemma/ flower and seed tissue | Predicted by Cell-PLoc 2.0 and WoLF PSORT software, expression profile studies | *Probably involved in transport of glucosinolates | Phylogenetic analysis (analogy with AtGTR1/2/3 transporters), RNA-Seq analysis, qRT-PCR | Glucosinolates | Wen et al. (2020) |
|  | *Brassica oleracea* var. *chinensis* Lei (Chinese kale) | BocGTR1a,  and BocGTR1c | NPF | *Leaves and buds | qRT-PCR | *Long-distance transport of glucosinolates from roots to leaves and stems | RNAi transgenic plant analysis, phylogenetic analysis, analogy with AtGTR transporters, HPLC analysis | Glucosinolates | Jiang et al. (2019) |
|  | *Brassica rapa*  (Mustard) | GTR1, and GTR2 | NPF | *Taproot | qRT-PCR, glucosinolate extraction | *Accumulation of indole glucosinolates in taproot | qRT-PCR, glucosinolate extraction | Glucosinolates | Touw et al. (2020) |
|  | *Catharanthus roseus*  (Periwinkle) | CrNPF2.9 | NPF | Tonoplast/leaf epidermis | YFP fusion study in suspension cultures, qRT-PCR | Export of strictosidine from leaf vacuole to cytosol | Expression in *Xenopus* oocytes, substrate uptake assays, virus-induced gene silencing, LC-MS analysis | Mono-  terpene indole alkaloids (MIA) | Payne et al. (2017) |
|  | *Catharanthus roseus*  (Periwinkle) | CrNPF2.4,  CrNPF2.5, and  CrNPF2.6 | NPF | Plasma membrane/leaf, stem, root, seedling | Infiltration of *N. benthamiana* leaves, GFP fusion, co-expression analysis | *Transport of iridoid glucoside and related compounds | Ortholog search using *A. thaliana* transporter library, expression and uptake activity assays in *Xenopus* oocytes | MIA precursor | Larsen et al. (2017b) |
| **Other transporters** | | | | | | | | | |
|  | *Catharanthus roseus*  (Periwinkle) | Proton/  alkaloid antiporter | - | *Tonoplast/  leaves | HPLC-DAD analysis of alkaloids in mesophyll protoplast and vacuole fractions | *Transport of vindoline from the cytosol to vacuole lumen | Vindoline uptake assays using tonoplast vesicles | Alkaloid | Carqueijeiro et al. (2013) |
|  | *Vitis vinifera*  (Grapevine) | BTL related protein | - | *Berry/epidermal tissue of pericarp | Light and fluorescence  Microscopy using berry sections | *Intercellular translocation of several anthocyanin precursors and other non-colored flavonoids | Immunoblot analysis and transport activity assay in microsomes | Flavonoid | Braidot et al. (2008) |

ABC: ATP binding cassette, AM: AnthoMATE, BUP: Benzylisoquinoline alkaloid uptake permease, BTL: Bilitranslocase, BY: Bright yellow, DAD: Diode array detector, FFT: Flower flavonoid transporter, GC: Gas chromatography, GFP: Green fluorescent protein, GUS: β-Glucuronidase, GTR: Glucosinolate transporter, HPLC: High performance liquid chromatography, JAT1: Jasmonate inducible alkaloid transporter 1, LC: Liquid chromatography, MATE: Multidrug and toxic compound extrusion, MDR: Multiple drug resistance, MRP: Multidrug resistance–associated protein, NPF: Nitrate/peptide transporter family, NUP: Nicotine uptake permease, PDR: Pleiotropic drug resistance, PUP: Purine uptake permease, RNAi: RNA interference, SSH: Suppression subtractive hybridization, TT12: Transparent testa 12, UHPLC-ESI-MS: Ultra high performance liquid chromatography electrospray ionization mass spectrometry, YFP: Yellow fluorescent protein

#The identification of probable transporters has majorly been done using bioinformatics tools and/or by comparatively analyzing with previously characterized transporters from other species. These studies still await more experimental validation.

*The exact localization and/or transport mechanism is either partially characterized based on preliminary/limited experimental information or indirect experiments have been used to draw conclusions.

**References**

Adebesin, F., Widhalm, J.R., Boachon, B., Lefevre, F., Pierman, B., Lynch, J.H., et al. (2017). Emission of volatile organic compounds from *Petunia* flowers is facilitated by an ABC transporter. *Science* 356(6345), 1386-1388. doi: 10.1126/science.aan0826

Alejandro, S., Lee, Y., Tohge, T., Sudre, D., Osorio, S., Park, J., et al. (2012). AtABCG29 is a monolignol transporter involved in lignin biosynthesis. *Curr. Biol.* 22(13), 1207-1212. doi: 10.1016/j.cub.2012.04.064

Andersen, T.G., Nour-Eldin, H.H., Fuller, V.L., Olsen, C.E., Burow, M., and Halkier, B.A. (2013). Integration of biosynthesis and long-distance transport establish organ-specific glucosinolate profiles in vegetative *Arabidopsis*. *Plant cell* 25(8), 3133-3145. doi: 10.1105/tpc.113.110890

Banasiak, J., Biala, W., Staszkow, A., Swarcewicz, B., Kepczynska, E., Figlerowicz, M., et al. (2013). A *Medicago truncatula* ABC transporter belonging to subfamily G modulates the level of isoflavonoids. *J. Exp. Bot.* 64(4), 1005-1015. doi: 10.1093/jxb/ers380

Behrens, C.E., Smith, K.E., Iancu, C.V., Choe, J.Y., Dean, J.V. (2019). Transport of anthocyanins and other flavonoids by the *Arabidopsis* ATP-binding cassette transporter AtABCC2. *Sci. Rep.* 9(437), 437. doi: 10.1038/s41598-018-37504-8

Biala, W., Banasiak, J., Jarzyniak, K., Pawela, A., and Jasinski, M. (2017). *Medicago truncatula* ABCG10 is a transporter of 4-coumarate and liquiritigenin in the medicarpin biosynthetic pathway. *J. Exp. Bot.* 68(12), 3231-3241. doi: 10.1093/jxb/erx059

Braidot, E., Petrussa, E., Bertolini, A., Peresson, C., Ermacora, P., Loi, N., et al. (2008). Evidence for a putative flavonoid translocator similar to mammalian bilitranslocase in grape berries (*Vitis vinifera* L.) during ripening. *Planta* 228(1), 203-213. doi: 10.1007/s00425-008-0730-4

Campbell, E.J., Schenk, P.M., Kazan, K., Penninckx, I.A., Anderson, J.P., Maclean, D.J., et al. (2003). Pathogen-responsive expression of a putative ATP-binding cassette transporter gene conferring resistance to the diterpenoid sclareol is regulated by multiple defense signaling pathways in *Arabidopsis*. *Plant Physiol.* 133(3), 1272-1284. doi: 10.1104/pp.103.024182.

Cao, H., Nuruzzaman, M., Xiu, H., Huang, J., Wu, K., Chen, X., et al. (2015). Transcriptome analysis of methyl jasmonate-elicited *Panax ginseng* adventitious roots to discover putative ginsenoside biosynthesis and transport genes. *Int. J. Mol. Sci.* 16(2), 3035-3057. doi: 10.3390/ijms16023035

Carqueijeiro, I., Noronha, H., Duarte, P., Geros, H., and Sottomayor, M. (2013). Vacuolar transport of the medicinal alkaloids from *Catharanthus roseus* is mediated by a proton-driven antiport. *Plant Physiol.* 162(3), 1486-1496. doi: 10.1104/pp.113.220558

Chang, X., Zhang, Z., Li, Y., Gao, J. (2018). Cloning and expression analyses of the mate gene in buckwheat. *Sci. Agric. Sin.* 51(11), 2038-2048. doi: 10.3864/j.issn.0578-1752.2018.11.002.

Chen, L., Liu, Y., Liu, H., Kang, L., Geng, J., Gai, Y., et al. (2015). Identification and expression analysis of MATE genes involved in flavonoid transport in blueberry plants. *PLoS One* 10(3), e0118578. doi: 10.1371/journal.pone.0118578

Chen, S.-Y., Tang, Y.-M., Hu, Y.-Y., Wang, Y., Sun, B., Wang, X.-R., et al. (2018). FaTT12-1 , a multidrug and toxin extrusion (MATE) member involved in proanthocyanidin transport in strawberry fruits. *Sci. Hortic.* 231, 158-165. doi: 10.1016/j.scienta.2017.12.032

Crouzet, J., Roland, J., Peeters, E., Trombik, T., Ducos, E., Nader, J., et al. (2013). NtPDR1, a plasma membrane ABC transporter from *Nicotiana tabacum*, is involved in diterpene transport. *Plant Mol. Biol.* 82(1-2), 181-192. doi: 10.1007/s11103-013-0053-0.

Dastmalchi, M., Chang, L., Chen, R., Yu, L., Chen, X., Hagel, J.M., et al. (2019). Purine permease-type benzylisoquinoline alkaloid transporters in opium poppy. *Plant Physiol.* 181(3), 916-933. doi: 10.1104/pp.19.00565

Debeaujon, I., Peeters, A.J.M., Léon-Kloosterziel, K.M., and Koornneef, M. (2001). The TRANSPARENT TESTA12 gene of *Arabidopsis* encodes a multidrug secondary transporter-like protein required for flavonoid sequestration in vacuoles of the seed coat endothelium. *Plant Cell* 13(4), 853. doi: 10.1105/tpc.13.4.853

Demurtas, O.C., Francisco, R., Diretto, G., Ferrante, P., Frusciante, S., Pietrella, et al. (2019). ABCC transporters mediate the vacuolar accumulation of crocins in saffron stigmas. *Plant Cell* 31, 2789-2804. doi: 10.1105/tpc.19.00193

Dong, B., Niu, L., Meng, D., Song, Z., Wang, L., Jian, Y., et al. (2019). Genome-wide analysis of MATE transporters and response to metal stress in *Cajanus cajan*. *J. Plant Interact.* 14(1), 265-275. doi: 10.1080/17429145.2019.1620884

Fourcroy, P., Sisó-Terraza, P., Sudre, D., Savirón, M., Reyt, G., Gaymard, F., et al. (2014). Involvement of the ABCG37 transporter in secretion of scopoletin and derivatives by *Arabidopsis* roots in response to iron deficiency. *New Phytol.* 201(1), 155-167. doi: 10.1111/nph.12471

Francisco, R.M., Regalado, A., Ageorges, A., Burla, B.J., Bassin, B., Eisenach, et al. (2013). ABCC1, an ATP binding cassette protein from grape berry, transports anthocyanidin 3-O-glucosides. *Plant Cell* 25(5), 1840-1854. doi: 10.1105/tpc.112.102152

Frank, S., Keck, M., Sagasser, M., Niehaus, K., Weisshaar, B., and Stracke, R. (2011). Two differentially expressed MATE factor genes from apple complement the *Arabidopsis* transparent testa12 mutant. *Plant Biol.* 13(1), 42-50. doi: 10.1111/j.1438-8677.2010.00350.x

Fu, X., Shi, P., He, Q., Shen, Q., Tang, Y., Pan, Q., et al. (2017). AaPDR3, a PDR transporter 3, is involved in sesquiterpene beta-caryophyllene transport in *Artemisia annua*. *Front. Plant Sci.* 8, 723. doi: 10.3389/fpls.2017.00723

Gao, J.S., Wu, N., Shen, Z.L., Lv, K., Qian, S.H., Guo, N., et al. (2016). Molecular cloning, expression analysis and subcellular localization of a Transparent Testa 12 ortholog in brown cotton (*Gossypium hirsutum* L.). *Gene* 576(2), 763-769. doi: 10.1016/j.gene.2015.11.002

Gomez, C., Terrier, N., Torregrosa, L., Vialet, S., Fournier-Level, A., Verries, C., et al. (2009). Grapevine MATE-type proteins act as vacuolar H^+^-dependent acylated anthocyanin transporters. *Plant Physiol.* 150(1), 402-415. doi: 10.1104/pp.109.135624

Goodman, C.D., Casati, P., and Walbot, V. (2004). A multidrug resistance-associated protein involved in anthocyanin transport in *Zea mays*. *Plant Cell* 16(7), 1812-1826. doi: 10.1105/tpc.022574

Hildreth, S.B., Gehman, E.A., Yang, H., Lu, R.H., Ritesh, K.C., Harich, K.C., et al. (2011). Tobacco nicotine uptake permease (NUP1) affects alkaloid metabolism. *Proc. Natl. Acad. Sci. U. S. A.* 108(44), 18179-18184. doi: 10.1073/pnas.1108620108

Jasiński, M., Stukkens, Y., Degand, H., Purnelle, B., Marchand-Brynaert, J., and Boutry, M. (2001). A plant plasma membrane ATP binding cassette–type transporter is involved in antifungal terpenoid secretion. *Plant cell* 13(5), 1095-1107. doi: 10.1105/tpc.13.5.1095

Jiang, D., Lei, J., Cao, B., Wu, S., Chen, G., and Chen, C. (2019). Molecular cloning and characterization of three glucosinolate transporter (GTR) genes from Chinese kale. *Genes* 10(3). doi: 10.3390/genes10030202

Jørgensen, M.E., Xu, D., Crocoll, C., Ernst, H.A., Ramírez, D., Motawia, et al. (2017). Origin and evolution of transporter substrate specificity within the NPF family. *eLife* 6, e19466. doi: 10.7554/eLife.19466

Kato, K., Shitan, N., Shoji, T., and Hashimoto, T. (2015). Tobacco NUP1 transports both tobacco alkaloids and vitamin B6. *Phytochemistry* 113, 33-40. doi: 10.1016/j.phytochem.2014.05.011

Khare, D., Choi, H., Huh, S.U., Bassin, B., Kim, J., Martinoia, E., et al. (2017). *Arabidopsis* ABCG34 contributes to defense against necrotrophic pathogens by mediating the secretion of camalexin. *Proc. Natl. Acad. Sci. U. S. A.* 114(28), E5712-E5720. doi: 10.1073/pnas.1702259114

Kitamura, S., Oono, Y., and Narumi, I. (2016). *Arabidopsis* pab1, a mutant with reduced anthocyanins in immature seeds from banyuls, harbors a mutation in the MATE transporter FFT. *Plant Mol. Biol.* 90(1-2), 7-18. doi: 10.1007/s11103-015-0389-8

Kretzschmar, T., Kohlen, W., Sasse, J., Borghi, L., Schlegel, M., Bachelier, et al. (2012). A *Petunia* ABC protein controls strigolactone-dependent symbiotic signalling and branching. *Nature* 483(7389), 341-344. doi: 10.1038/nature10873

Larsen, B., Fuller, V.L., Pollier, J., Van Moerkercke, A., Schweizer, F., Payne, R., et al. (2017b). Identification of iridoid glucoside transporters in *Catharanthus roseus*. *Plant Cell Physiol.* 58(9), 1507-1518. doi: 10.1093/pcp/pcx097

Lopez-Ortiz, C., Dutta, S.K., Natarajan, P., Pena-Garcia, Y., Abburi, V., Saminathan, T., et al. (2019). Genome-wide identification and gene expression pattern of ABC transporter gene family in *Capsicum* spp. *PLoS One* 14(4), e0215901. doi: 10.1371/journal.pone.0215901

M’mbone, M.E., Cheng, W., Xu, L., Wang, Y., Karanja, B.K., Zhu, X., et al. (2018). Identification and transcript analysis of MATE genes involved in anthocyanin transport in radish (*Raphanus sativus* L.). *Sci. Hortic.* 238, 195-203. doi: 10.1016/j.scienta.2018.04.029

Mackon, E., Ma, Y., Jeazet Dongho Epse Mackon, G.C., Usman, B., Zhao, Y., Li, Q., et al. (2021). Computational and transcriptomic analysis unraveled OsMATE34 as a putative anthocyanin transporter in Black Rice (*Oryza sativa* L.) caryopsis. *Genes* 12(4). doi: 10.3390/genes12040583

Mathews, H., Clendennen, S.K., Caldwell, C.G., Liu, X.L., Connors, K., Matheis, N., et al. (2003). Activation tagging in tomato identifies a transcriptional regulator of anthocyanin biosynthesis, modification, and transport. *Plant Cell* 15(8), 1689-1703. doi: 10.1105/tpc.012963

Miao, G., Han, J., Huo, Y.B., Wang, C.R., Wang, S.C. (2021). Identification and functional characterization of a PDR transporter in *Tripterygium wilfordii* Hook.f. that mediates the efflux of triptolide. *Plant Mol. Biol.* 106(1-2), 145-156. doi: 10.1007/s11103-021-01134-y

Morita, M., Shitan, N., Sawada, K., Van Montagu, M.C.E., Inze, D., Rischer, H., et al. (2009). Vacuolar transport of nicotine is mediated by a multidrug and toxic compound extrusion (MATE) transporter in *Nicotiana tabacum*. *Proc. Natl. Acad. Sci. U. S. A.* 106(7), 2447-2452. doi: 10.1073/pnas.0812512106

Nambiar, D.M., Kumari, J., Augustine, R., Kumar, P., Bajpai, P.K., and Bisht, N.C. (2021). GTR1 and GTR2 transporters differentially regulate tissue-specific glucosinolate contents and defence responses in the oilseed crop *Brassica juncea*. *Plant Cell Environ.* 1-15. doi: 10.1111/pce.14072

Nour-Eldin, H.H., Andersen, T.G., Burow, M., Madsen, S.R., Jorgensen, M.E., Olsen, C.E., et al. (2012). NRT/PTR transporters are essential for translocation of glucosinolate defence compounds to seeds. *Nature* 488(7412), 531-534. doi: 10.1038/nature11285

Payne, R.M., Xu, D., Foureau, E., Teto Carqueijeiro, M.I., Oudin, A., Bernonville, T.D., et al. (2017). An NPF transporter exports a central monoterpene indole alkaloid intermediate from the vacuole. *Nat. Plants* 3, 16208. doi: 10.1038/nplants.2016.208

Perez-Diaz, R., Ryngajllo, M., Perez-Diaz, J., Pena-Cortes, H., Casaretto, J.A., Gonzalez-Villanueva, E., et al. (2014). VvMATE1 and VvMATE2 encode putative proanthocyanidin transporters expressed during berry development in *Vitis vinifera* L. *Plant Cell Rep.* 33(7), 1147-1159. doi: 10.1007/s00299-014-1604-9

Pierman, B., Toussaint, F., Bertin, A., Levy, D., Smargiasso, N., De Pauw, E., et al. (2017). Activity of the purified plant ABC transporter NtPDR1 is stimulated by diterpenes and sesquiterpenes involved in constitutive and induced defenses. *J. Biol. Chem.* 292(47), 19491-19502. doi: 10.1074/jbc.M117.811935

Ruocco, M., Ambrosino, P., Lanzuise, S., Woo, S.L., Lorito, M., and Scala, F. (2011). Four potato (*Solanum tuberosum*) ABCG transporters and their expression in response to abiotic factors and *Phytophthora infestans* infection. *J. Plant. Physiol.* 168(18), 2225-2233. doi: 10.1016/j.jplph.2011.07.008

Ruzicka, K., Strader, L.C., Bailly, A., Yang, H., Blakeslee, J., Langowski, L., et al. (2010). *Arabidopsis* PIS1 encodes the ABCG37 transporter of auxinic compounds including the auxin precursor indole-3-butyric acid. *Proc. Natl. Acad. Sci. U. S. A.* 107(23), 10749-10753. doi: 10.1073/pnas.1005878107

Sasabe, M., Toyoda, K., Shiraishi, T., Inagaki, Y., Ichinose, Y. (2002). cDNA cloning and characterization of tobacco ABC transporter: NtPDR1 is a novel elicitor-responsive gene 1. *FEBS Lett.* 518(1-3), 164-168. doi: 10.1016/s0014-5793(02)02697-2.

Shibata, Y., Ojika, M., Sugiyama, A., Yazaki, K., Jones, D.A., Kawakita, K., et al. (2016). The full-size ABCG transporters Nb-ABCG1 and Nb-ABCG2 function in pre- and postinvasion defense against *Phytophthora infestans* in *Nicotiana benthamiana*. *Plant Cell* 28(5), 1163-1181. doi: 10.1105/tpc.15.00721

Shitan, N., Bazin, I., Dan, K., Obata, K., Kigawa, K., Ueda, K., et al. (2003). Involvement of CjMDR1, a plant multidrug-resistance-type ATP-binding cassette protein, in alkaloid transport in *Coptis japonica*. *Proc. Natl. Acad. Sci. U. S. A.* 100(2), 751-756. doi: 10.1073/pnas.0134257100

Shitan, N., Dalmas, F., Dan, K., Kato, N., Ueda, K., Sato, F., et al. (2013). Characterization of *Coptis japonica* CjABCB2, an ATP-binding cassette protein involved in alkaloid transport. *Phytochemistry* 91, 109-116. doi: 10.1016/j.phytochem.2012.02.012

Shitan, N., Hayashida, M., and Yazaki, K. (2015). Translocation and accumulation of nicotine via distinct spatio-temporal regulation of nicotine transporters in *Nicotiana tabacum*. *Plant Signal. Behav.* 10(7), e1035852. doi: 10.1080/15592324.2015.1035852

Shitan, N., Minami, S., Morita, M., Hayashida, M., Ito, S., Takanashi, K., et al. (2014). Involvement of the leaf-specific multidrug and toxic compound extrusion (MATE) transporter Nt-JAT2 in vacuolar sequestration of nicotine in *Nicotiana tabacum*. *PLoS One* 9(9), e108789. doi: 10.1371/journal.pone.0108789

Shitan, N., Morita, M., and Yazaki, K. (2009). Identification of a nicotine transporter in leaf vacuoles of *Nicotiana tabacum*. *Plant Signal. Behav.* 4(6), 530-532. doi: 10.4161/psb.4.6.8588

Shoji, T., Inai, K., Yazaki, Y., Sato, Y., Takase, H., Shitan, N., et al. (2009). Multidrug and toxic compound extrusion-type transporters implicated in vacuolar sequestration of nicotine in tobacco roots. *Plant Physiol.* 149(2), 708-718. doi: 10.1104/pp.108.132811

Stukkens, Y., Bultreys, A., Grec, S., Trombik, T., Vanham, D., and Boutry, M. (2005). NpPDR1, a pleiotropic drug resistance-type ATP-binding cassette transporter from *Nicotiana plumbaginifolia*, plays a major role in plant pathogen defense. *Plant Physiol.* 139(1), 341-352. doi: 10.1104/pp.105.062372.

Sun, N., Xie, Y.F., Wu, Y., Guo, N., Li, D.H., and Gao, J.S. (2021). Genome-wide identification of ABCC gene family and their expression analysis in pigment deposition of fiber in brown cotton (*Gossypium hirsutum*). *PLoS One* 16(5), e0246649. doi: 10.1371/journal.pone.0246649

Takanashi, K., Yamada, Y., Sasaki, T., Yamamoto, Y., Sato, F., and Yazaki, K. (2017). A multidrug and toxic compound extrusion transporter mediates berberine accumulation into vacuoles in *Coptis japonica*. *Phytochemistry* 138, 76-82. doi: 10.1016/j.phytochem.2017.03.003

Touw, A.J., Verdecia Mogena, A., Maedicke, A., Sontowski, R., van Dam, N.M., and Tsunoda, T. (2020). Both biosynthesis and transport are involved in glucosinolate accumulation during root-herbivory in *Brassica rapa*. *Front. Plant Sci.* 10, 1653. doi: 10.3389/fpls.2019.01653

van den Brule, S., Muller, A., Fleming, A.J., and Smart, C.C. (2002). The ABC transporter SpTUR2 confers resistance to the antifungal diterpene sclareol. *Plant J.* 30(6), 649-662. doi: 10.1046/j.1365-313x.2002.01321.x

Wen, J., Li, P.F., Ran, F., Guo, P.C., Zhu, J.T., Yang, J., et al. (2020). Genome-wide characterization, expression analyses, and functional prediction of the NPF family in *Brassica napus*. *BMC Genomics* 21(1), 871. doi: 10.1186/s12864-020-07274-7

Xie, X., Wang, G., Yang, L., Cheng, T., Gao, J., Wu, Y., et al. (2015). Cloning and characterization of a novel *Nicotiana tabacum* ABC transporter involved in shoot branching. *Physiol. Plant* 153(2), 299-306. doi: 10.1111/ppl.12267

Xu, L., Shen, Z.L., Chen, W., Si, G.Y., Meng, Y., Guo, N., et al. (2019). Phylogenetic analysis of upland cotton MATE gene family reveals a conserved subfamily involved in transport of proanthocyanidins. *Mol. Biol. Rep.* 46(1), 161-175. doi: 10.1007/s11033-018-4457-4

Yan, L., Zhang, J., Chen, H., and Luo, H. (2021). Genome-wide analysis of ATP-binding cassette transporter provides insight to genes related to bioactive metabolite transportation in *Salvia miltiorrhiza*. *BMC Genomics* 22(1), 315. doi: 10.21203/rs.3.rs-99773/v1.

Yazaki, K., Shitan, N., Takamatsu, H., Ueda, K., and Sato, F. (2001). A novel *Coptis japonica* multidrug‐resistant protein preferentially expressed in the alkaloid‐accumulating rhizome. *J. Exp. Bot.* 52(357), 877-879. doi: 10.1093/jexbot/52.357.877

Yu, F., and De Luca, V. (2013). ATP-binding cassette transporter controls leaf surface secretion of anticancer drug components in *Catharanthus roseus*. *Proc. Natl. Acad. Sci. U. S. A.* 110(39), 15830-15835. doi: 10.1073/pnas.1307504110

Zhang, R., Huang, J., Zhu, J., Xie, X., Tang, Q., Chen, X., et al. (2013). Isolation and characterization of a novel PDR-type ABC transporter gene PgPDR3 from *Panax ginseng* C.A. Meyer induced by methyl jasmonate. *Mol. Biol. Rep.* 40(11), 6195-6204. doi: 10.1007/s11033-013-2731-z

Zhao, J., and Dixon, R.A. (2009). MATE transporters facilitate vacuolar uptake of epicatechin 3′-O-glucoside for proanthocyanidin biosynthesis in *Medicago truncatula* and *Arabidopsis*. *Plant Cell* 21(8), 2323-2340. doi: 10.1105/tpc.109.067819

Zhao, J., Huhman, D., Shadle, G., He, X.Z., Sumner, L.W., Tang, Y., et al. (2011). MATE2 mediates vacuolar sequestration of flavonoid glycosides and glycoside malonates in *Medicago truncatula*. *Plant Cell* 23(4), 1536-1555. doi: 10.1105/tpc.110.080804

Zhong, F., Huang, L., Qi, L., Ma, Y., and Yan, Z. (2020). Full-length transcriptome analysis of *Coptis deltoidea* and identification of putative genes involved in benzylisoquinoline alkaloids biosynthesis based on combined sequencing platforms. *Plant Mol. Biol.* 102(4-5), 477-499. doi: 10.1007/s11103-019-00959-y
